# Optimization of scarless human stem cell genome editing

**DOI:** 10.1093/nar/gkt555

**Published:** 2013-07-31

**Authors:** Luhan Yang, Marc Guell, Susan Byrne, Joyce L. Yang, Alejandro De Los Angeles, Prashant Mali, John Aach, Caroline Kim-Kiselak, Adrian W Briggs, Xavier Rios, Po-Yi Huang, George Daley, George Church

**Affiliations:** ^1^Department of Genetics, Harvard Medical School, Boston, 02115 MA, USA, ^2^Biological and Biomedical Sciences Program, Harvard Medical School, Boston, 02115 MA, USA, ^3^Children’s Hospital, Boston, 02115 MA, USA, ^4^Chemistry and Chemical Biology program, Harvard, 02138 Cambridge, MA, USA and ^5^Wyss Institute for Biologically Inspired Engineering, Harvard University, Cambridge, 02138 MA, USA

## Abstract

Efficient strategies for precise genome editing in human-induced pluripotent cells (hiPSCs) will enable sophisticated genome engineering for research and clinical purposes. The development of programmable sequence-specific nucleases such as Transcription Activator-Like Effectors Nucleases (TALENs) and Cas9-gRNA allows genetic modifications to be made more efficiently at targeted sites of interest. However, many opportunities remain to optimize these tools and to enlarge their spheres of application. We present several improvements: First, we developed functional re-coded TALEs (reTALEs), which not only enable simple one-pot TALE synthesis but also allow TALE-based applications to be performed using lentiviral vectors. We then compared genome-editing efficiencies in hiPSCs mediated by 15 pairs of reTALENs and Cas9-gRNA targeting *CCR5* and optimized ssODN design in conjunction with both methods for introducing specific mutations. We found Cas9-gRNA achieved 7–8× higher non-homologous end joining efficiencies (3%) than reTALENs (0.4%) and moderately superior homology-directed repair efficiencies (1.0 versus 0.6%) when combined with ssODN donors in hiPSCs. Using the optimal design, we demonstrated a streamlined process to generated seamlessly genome corrected hiPSCs within 3 weeks.

## INTRODUCTION

Precise genome editing in human-induced pluripotent cells (hiPSCs) will enable functional studies of human genetic variation and enhance the potential use of hiPSCs for regenerative medicine. Currently, genome editing via sequence-specific nucleases represents the most efficient way to precisely edit human cell genomes (1–3). A nuclease-mediated double-stranded DNA (dsDNA) break in the genome can be repaired by two main mechanisms (4): non-homologous end joining (NHEJ), which frequently results in the introduction of non-specific insertions and deletions (indels), or homology-directed repair (HDR), which incorporates a homologous strand as a repair template. When a sequence-specific nuclease is delivered along with a homologous donor DNA construct containing the desired mutations, gene targeting efficiencies are increased by 1000-fold compared with just the donor construct alone (5). Thus, the development of programmable nucleases has greatly facilitated the practice of targeted genome engineering.

Despite large advances in gene editing tools, many challenges and questions remain regarding the use of custom-engineered nucleases in hiPSC engineering. First, despite their design simplicity, Transcription Activator-Like Effectors Nucleases (TALENs) target particular DNA sequences with tandem copies of Repeat Variable Diresidue (RVD) domains (6). Although the modular nature of RVDs simplifies TALEN design, their repetitive sequences complicate methods for synthesizing their DNA constructs (7–10) and also impair their use with lentiviral gene delivery vehicles, most likely by causing sequence instabilities (11).

Next, we sought to improve the ease and sensitivity of current detection methods for assessing genome editing. In current practice, NHEJ and HDR are frequently evaluated using separate assays. Mismatch-sensitive endonuclease assays (12) are often used for assessing NHEJ, but the quantitative accuracy of this method is variable, and the sensitivity is limited to NHEJ frequencies greater than ∼3% (12). Meanwhile, HDR is frequently assessed by cloning and sequencing, a completely different and often cumbersome procedure. Sensitivity is still an issue because, although high editing frequencies on the order of 50% are frequently reported for some cell types, such as U2OS and K562 (10,13), frequencies are generally lower in hiPSCs (14). Recently, high editing frequencies have been reported in hiPSC and hESC using TALENs (15) and even higher frequencies with the CRISPR Cas9-gRNA system (16–19). However, editing rates at different sites appear to vary widely (17), and editing is sometimes not detectable at all at some sites (20). Moreover, although the recent successes in editing hiPSC genomes with TALENs and Cas9 are striking, genome editing using these tools has not yet been systematically explored and compared. To come to a fuller understanding of these issues and optimize inefficiencies will require simple and efficient collection and analysis of NHEJ and HDR rates at large numbers of sites using tools that accurately capture low as well as high rates. To this end, we developed a robust and user-friendly package using next generation sequencing to screen HR and NHEJ events in hiPSCs together.

As a demonstration of how our improved synthesis method for TALEs, and our genome editing assessment tool, can expedite data gathering, analysis and optimization, we used these tools to compare reTALEN and Cas9 efficiencies in hiPSCs at 15 sites near the *CCR5* locus. As with TALEN and Cas9 editing of hiPSCs, generally, use of ssODNs as DNA donors has been reported (21,22), but the optimal design and scope of ssODNs for this purpose have not been systematically explored. We then used our tools to optimize the design of ssODNs used as donors for scarless genome engineering.

Another area for improvement in editing procedures for hiPSC relates to the clonal isolation of the hiPSCs themselves, an operation that is difficult in part because hiPSC are difficult to grow out from isolated single cells because in the absence of appropriate cell-to-cell contacts with other hiPSCs or feeder cells. However, procedures that improve clonal hiPSC isolation have recently been reported (23), and we adapted these to integrate with the other procedures we report here. Taken all together, we demonstrate that it is possible to obtain clonal, precisely genome-edited hiPSCs within 3 weeks, including within this the amount of time required to synthesize optimal reagents and perform rapid prospective screening of target events.

## MATERIALS AND METHODS

### gRNA assembly

We incorporated 19 bp of the selected target sequence (i.e. 5′-N_19_ of 5′-N_19_-NGG-3′) into two complementary 100 mer oligonucleotides (TTCTTGGCTTTATATATCTTGTGGAAAGGACGAAACACCGN19GTTTTAGAGCTAGAAATAGCAAGTTAAAATAAGGCTAGTCC). Each 100 mer oligonucleotide was suspended at 100 mM in water, mixed with equal volume and annealed in thermocycle machine (95°C, 5 min; Ramp to 4°C, 0.1°C/s). To prepare the destination vector, we linearized the gRNA cloning vector (Addgene plasmid ID 41824, Supplementary Sequence S3) using AfIII and purified the vector through purification. We carried out the (10 µl) gRNA assembly reaction with 10 ng annealed 100 bp fragment, 100 ng destination backbone, 1× Gibson assembly reaction mix (New England Biolabs) at 50°C for 30 min, and reaction can be processed directly for bacterial transformation to colonize individual assemblies.

### re-TALEs design and assembly

re-TALEs were optimized at different levels to facilitate assembly and improve expression. re-TALE DNA sequences were first co-optimized for a human codon-usage and low mRNA folding energy at the 5′ end (GeneGA, Bioconductor). The obtained sequence was evolved through several cycles to eliminate repeats (direct or inverted) longer than 11 bp (Supplementary Figure S8). In each cycle, synonymous sequences for each repeat are evaluated. Those with the largest hamming distance to the evolving DNA are selected. The sequence of one of re-TALE possessing 16.5 monomers is listed in Supplementary Sequence S1.

re-TALE dimer blocks encoding two RVDs (Supplementary Figure S2A) were generated by two rounds of PCR under standard Kapa HIFI (KPAP) PCR conditions, in which the first round of PCR introduced the RVD coding sequence and the second round of PCR generated the entire dimer blocks with 36 bp overlaps with the adjacent blocks. PCR products were purified using QIAquick 96 PCR Purification Kit (QIAGEN), and the concentrations were measured by Nano-drop. The primer and template sequences are listed in Supplementary Tables S1 and S2.

re-TALENs and re-TALE-TF destination vectors were constructed by modifying the TALE-TF and TALEN cloning backbones (24). We re-coded the 0.5 RVD regions on the vectors and also incorporated SapI cutting site at the designated re-TALE cloning site. The sequences of re-TALENs and re-TALE-TF backbones are listed in Supplementary Sequence S2. Plasmids can be pre-treated with SapI (New England Biolabs) with manufacturer recommended conditions and purified with QIAquick PCR purification kit (QIAGEN).

We carried out the (10 µl) one-pot TALE Single-incubation Assembly (TASA) assembly reaction with 200 ng of each block, 500 ng of destination backbone, 1× TASA enzyme mixture [2U SapI, 100 U Ampligase (Epicentre), 10 mU T5 exonuclease (Epicentre), 2.5U Phusion DNA polymerase (New England Biolabs)] and 1× isothermal assembly reaction buffer as described before (25) [5% PEG-8000, 100 mM Tris–HCl (pH 7.5), 10 mM MgCl2, 10 mM DTT, 0.2 mM each of the four dNTPs and 1 mM NAD]. Incubations were performed at 37°C for 5 min and 50°C for 30 min. TASA assembly reaction can be processed directly for bacterial transformation to colonize individual assemblies. The efficiency of obtaining full-length construct is ∼20% with this approach. Alternatively, >90% efficiency can be achieved by three-steps assembly. First, 10 µl of re-TALE assembly reactions were performed with 200 ng of each block, 1× re-TALE enzyme mixture (100 U Ampligase, 12.5 mU T5 exonuclease, 2.5 U Phusion DNA polymerase) and 1× isothermal assembly buffer at 50°C for 30 min, followed by standardized Kapa HIFI PCR reaction, agarose gel electrophoresis and QIAquick Gel extraction (Qiagen) to enrich the full-length re-TALEs. In all, 200 ng of re-TALE amplicons can then be mixed with 500 ng of Sap1-pre-treated destination backbone, 1× re-TALE assembly mixture and 1× isothermal assembly reaction buffer and incubated at 50°C for 30 min. The re-TALE final assembly reaction can be processed directly for bacterial transformation to colonize individual assemblies. Additional notes of the assembly methods can be found in Supplementary Note S1.

### Cell line and cell culture

PGP1 iPS cells were maintained on Matrigel (BD Biosciences)-coated plates in mTeSR1 (Stemcell Technologies). Cultures were passaged every 5–7 days with TrypLE Express (Invitrogen). The 293 T and 293FT cells were grown and maintained in Dulbecco’s modified Eagle’s medium (DMEM, Invitrogen) high glucose supplemented with 10% fetal bovine serum (Invitrogen), penicillin/streptomycin (pen/strep, Invitrogen) and non-essential amino acids (Invitrogen). K562 cells were grown and maintained in RPMI (Invitrogen) supplemented with 10% fetal bovine serum (Invitrogen 15%) and penicillin/streptomycin (pen/strep, Invitrogen). All cells were maintained at 37°C and 5% CO2 in a humidified incubator.

We established a stable 293T cell line for detecting HDR efficiency as described before (26). Specifically, the reporter cell lines bear genomically integrated GFP-coding sequences disrupted by the insertion of a stop codon and a 68 bp genomic fragment derived from the AAVS1 locus.

### Test of reTALENs activity

We seeded 293 T reporter cells at densities of 2 × 105 cells per well in 24-well plate and transfected them with 1 μg of each re-TALENs plasmid and 2 μg DNA donor plasmid using Lipofectamine 2000 following the manufacturer’s protocols. Cells were harvested using TrypLE Express (Invitrogen) ∼18 h after transfection and resuspended in 200 µl of media for flow cytometry analysis using an LSRFortessa cell analyzer (BD Biosciences). The flow cytometry data were analyzed using FlowJo (FlowJo). At least 25 000 events were analyzed for each transfection sample. For endogenous AAVS1 locus targeting experiment in 293 T, the transfection procedures were identical as described earlier in the text, and we conducted puromycin selection with drug concentration at 3 μg/ml 1 week after transfection.

### Functional lentivirus generation assessment

The lentiviral vectors were created by standard PCR and cloning techniques. The lentiviral plasmids were transfected by Lipofectamine 2000 with Lentiviral Packaging Mix (Invitrogen) into cultured 293FT cells (Invitrogen) to produce lentivirus. Supernatant was collected 48 and 72 h post-transfection, sterile filtered and 100 µl of filtered supernatant was added to 5 × 10^5^ fresh 293 T cells with polybrene. Lentivirus titration was calculated based on the following formula: virus titration = (percentage of GFP+ 293 T cell × initial cell numbers under transduction)/(the volume of original virus collecting supernatant used in the transduction experiment). To test the functionality of lentivirus, 3 days after transduction, we transfected lentivirus transduced 293 T cells with 30 ng of plasmids carrying mCherry reporter and 500 ng of pUC19 plasmids using Lipofectamine 2000 (Invitrogen). Cell images were analyzed using Axio Observer Z.1 (Zeiss) 18 h after transfection and harvested using TrypLE Express (Invitrogen) and resuspended in 200 µl of media for flow cytometry analysis using LSRFortessa cell analyzer (BD Biosciences). The flow cytometry data were analyzed using BD FACSDiva (BD Biosciences).

### Test of re-TALENs and Cas9-gRNA genome editing efficiency

PGP1 iPSCs were cultured in Rho kinase (ROCK) inhibitor Y-27632 (Calbiochem) 2 h before nucleofection. Transfections were done using P3 Primary Cell 4D-Nucleofector X Kit (Lonza). Specifically, cells were harvested using TrypLE Express (Invitrogen), and 2 × 10^6^ cells were resuspended in 20 μl of nucleofection mixture containing 16.4 μl of P3 Nucleofector solution, 3.6 μl of supplement, 1 μg of each re-TALENs plasmid or 1 µg of Cas9 and 1 µg of gRNA construct, 2 μl of 100 μM ssODN. Subsequently, we transferred the mixtures to 20 µl of Nucleocuvette strips and conducted nucleofection using CB150 program. Cells were plated on Matrigel-coated plates in mTeSR1 medium supplemented with ROCK inhibitor for the first 24 h. For endogenous AAVS1 locus-targeting experiment with dsDNA donor, we used the identical procedure except we used 2 μg of dsDNA donor, and we supplement the mTeSR1 media with puromycin at the concentration of 0.5 µg/ml 1 week after transfection.

The information of reTALENs, gRNA and ssODNs used in this study are listed in Supplementary Tables S3 and S6.

### Amplicon library preparation of the targeting regions

Cells were harvested 6 days after nucleofection and 0.1 μl of prepGEM tissue protease enzyme (ZyGEM) and 1 μl of prepGEM gold buffer (ZyGEM) were added to 8.9 µl of the 2–5 × 10^5^ cells in the medium. In all, 1 µl of the reactions were then added to 9 µl of PCR mix containing 5 µl 2 × KAPA Hifi Hotstart Readymix (KAPA Biosystems) and 100 nM corresponding amplification primer pairs. Reactions were incubated at 95°C for 5 min followed by 15 cycles of 98°C, 20 s; 65°C, 20 s and 72°C, 20 s. To add the Illumina sequence adaptor, 5 µl of reaction products were then added to 20 µl of PCR mix containing 12.5 µl of 2 × KAPA HIFI Hotstart Readymix (KAPA Biosystems) and 200 nM primers carrying Illumina sequence adaptors. Reactions were incubated at 95°C for 5 min followed by 25 cycles of 98°C, 20 s; 65°C, 20 s and 72°C, 20 s. PCR products were purified by QIAquick PCR purification kit, mixed at roughly the same concentration and sequenced with MiSeq Personal Sequencer. All the PCR primers can be found in the Supplementary Table S5.

### Genome editing assessment system

We wrote a pipeline to analyze the genome engineering data. This pipeline is integrated in one single Unix module, which uses different tools such as R, BLAT and FASTX Toolkit.

Barcode splitting: Groups of samples were pooled together and sequenced using MiSeq 150 bp paired end (PE150) (Illumina Next Gen Sequencing) and later separated based on DNA barcodes using FASTX Toolkit.

Quality filtering: We trimmed nucleotides with lower sequence quality (phred score <20). After trimming, reads shorter than 80 nt were discarded.

Mapping: We used BLAT to map the paired reads independently to the reference genome and we generated .psl files as output.

Indel calling: We defined indels as the full-length reads containing two blocks of matches in the alignment. Only reads following this pattern in both paired end reads were considered. As a quality control, we required the indel reads to possess minimal 70 nt matching with the reference genome and both blocks to be at least 20 nt long. Size and position of indels were calculated by the positions of each block to the reference genome. Non-homologous end joining (NHEJ) has been estimated as the percentage of reads containing indels [see Equation (1)]. The majority of NHEJ event have been detected at the targeting site vicinity.

Homology-directed recombination (HDR) efficiency: Pattern matching (grep) within a 12 bp window centering over DSB was used to count specific signatures corresponding to reads containing the reference sequence, modifications of the reference sequence (2 bp intended mismatches) and reads containing only 1 bp mutation within the 2 bp intended mismatches [see Equation (1)].

Equation 1. Estimation of NHEJ and HDR

A=reads identical to the reference: XXXXXABXXXXX

B =reads containing 2 bp mismatch programed by ssODN: XXXXXabXXXXX

C = reads containing only 1 bp mutation in the target site: such as XXXXXaBXXXXX or XXXXXAbXXXXX

D = reads containing indels as described above

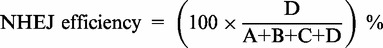


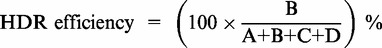



The statistic analysis of the GEAS can be found in Supplementary Note S2.

### Genotype screening of colonized hiPSCs

Human iPS cells on feeder-free cultures were pre-treated with mTesr-1 media supplemented with SMC4 (5 uM thiazovivin, 1 uM CHIR99021, 0.4 uM PD0325901, 2 uM SB431542) (23) for at least 2 h before fluorescence-activated cell sorting (FACS) sorting. Cultures were dissociated using Accutase (Millipore) and resuspended in mTesr-1 media supplemented with SMC4 and the viability dye ToPro-3 (Invitrogen) at concentration of 1–2 × 107 /ml. Live hiPS cells were single-cell sorted using a BD FACSAria II SORP UV (BD Biosciences) with 100 µm nozzle under sterile conditions into 96-well plates coated with irradiated CF-1 mouse embryonic fibroblasts (Global Stem). Each well contained hES cell medium (27) with 100 ng/ml recombinant human basic Fibroblast Growth Factor (Millipore) supplemented with SMC4 and 5 µg/ml fibronectin (Sigma). After sorting, plates were centrifuged at 70*g* for 3 min. Colony formation was seen 4 days post sorting, and the culture media was replaced with hES cell medium with SMC4. SMC4 can be removed from hES cell medium 8 days after sorting.

A few thousand cells were harvested 8 days after FACS and 0.1 µl of prepGEM tissue protease enzyme (ZyGEM) and 1 µl of prepGEM gold buffer (ZyGEM) were added to 8.9 µl of cells in the medium. The reactions were then added to 40 µl of PCR mix containing 35.5 ml of platinum 1.1× Supermix (Invitrogen), 250 nM of each dNTP and 400 nM primers. Reactions were incubated at 95°C for 3 min followed by 30 cycles of 95°C, 20 s; 65°C, 30 s and 72°C, 20 s. Products were Sanger sequenced using either one of the PCR primers (Supplementary Table S5), and sequences were analyzed using DNASTAR (DNASTAR).

### Immunostaining and teratoma assays of hiPSCs

Cells were incubated in the KnockOut DMEM/F-12 medium at 37°C for 60 min using the following antibody: Anti-SSEA-4 PE (Millipore) (1: 500 diluted); Tra-1-60 (BD Pharmingen) (1:100 diluted). After the incubation, cells were washed three times with KnockOut DMEM/F-12 and imaged on the Axio Observer Z.1 (ZIESS).

To conduct teratoma formation analysis, we harvested human iPSCs using collagenase type IV (Invitrogen) and resuspended the cells into 200 µl of Matrigel and injected intramuscularly into the hind limbs of Rag2gamma knockout mice. Teratomas were isolated and fixed in formalin between 4 and 8 weeks after the injection. The teratomas were subsequently analyzed by hematoxylin and eosin staining.

## RESULTS

### ReTALENs target genomic loci effectively in human somatic and stem cells

TALEs have proven to be a powerful and easy-to-design tool for targeted genome manipulation in multiple cell lines and organisms (2,13–15, 28–30). Several strategies have been developed to assemble the repetitive TALE RVD array sequences (7–10). However, once assembled, the TALE sequence repeats remain unstable, which limits the wide utility of this tool, especially for viral gene delivery vehicles (11,31). We thus thought that complete elimination of repeats would not only enable faster and simple synthesis of extended TALE RVD arrays but also address this important post-synthesis problem.

To eliminate repeats, we computationally evolved the nucleotides sequence of TALE RVD arrays to minimize the number of sequence repeats while maintaining the amino acid composition. Re-coded TALE (Re-TALEs) encoding 16 tandem RVD DNA recognition monomers, plus the final half RVD repeat, are devoid of any 12 bp repeats (Supplementary Figure S1a). Notably, this level of recoding is sufficient to allow PCR amplification of any specific monomer or sub-section from a full-length re-TALE construct (Supplementary Figure S1b). The improved design of re-TALEs makes it possible to order them directly from gene synthesis companies using standard DNA synthesis technology (32), without incurring the additional costs or procedures associated with repeat-heavy sequences. Furthermore, the recoded sequence design also enabled us to efficiently assemble re-TALE constructs using a modified isothermal assembly reaction (‘Materials and Methods’ section, Supplementary Note S1, Supplementary Figure S2).

**Figure 1.**
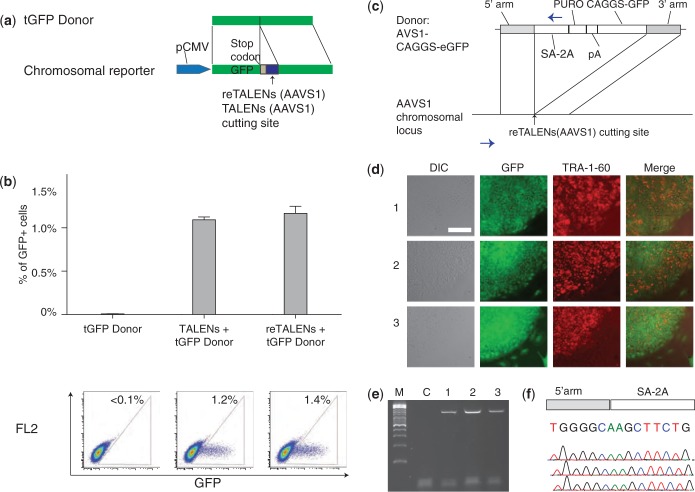
Functional tests of re-TALENs in human somatic and stem cells. (**a**) Schematic representation of experimental design for testing genome targeting efficiency. A genomically integrated GFP-coding sequence is disrupted by the insertion of a stop codon and a 68 bp genomic fragment derived from the AAVS1 locus (bottom). Restoration of the GFP sequence by nuclease-mediated homologous recombination with tGFP donor (top) results in GFP+ cells that can be quantitated by FACS. Re-TALENs and TALENs target identical sequences within AAVS1 fragments. (**b**) Bar graph depicting GFP+ cell percentage introduced by tGFP donor alone, TALENs with tGFP donor and re-TALENs with tGFP donor at the target locus, as measured by FACS (*N* = 3, error bar = SD). Representative FACS plots are shown later in the text. (**c**) Schematic overview depicting the targeting strategy for the native AAVS1 locus. The donor plasmid, containing splicing acceptor (SA)- 2 A (self-cleaving peptides), puromycin resistant gene (PURO) and GFP were described before (14). The locations of PCR primers used to detect successful editing events are depicted as blue arrows. (**d**) Successfully targeted clones of PGP1 hiPSCs were selected with puromycin (0.5 µg/ml) for 2 weeks. Microscopy images of three representative GFP+ clones are shown. Cells were also stained for the pluripotency markers TRA-1-60. Scale bar: 200 µm. (**e**) PCR assays performed on these the monoclonal GFP+ hiPSC clones demonstrated successful insertions of the donor cassettes at the AAVS1 site (lanes 1–3), whereas plain hiPSCs show no evidence of successful insertion (lane C). (**f**) Sanger sequencing of the PCR amplicon from the three targeted hiPSC colonies confirmed that the expected DNA bases at the genome-insertion boundary is present.

We next sought to test the function of reTALEN in comparison with the corresponding non-recoded TALEN in human cells. To this end, we used a HEK 293 cell line containing a GFP reporter cassette carrying a frame-shifting insertion as previously described (33) (Figure 1a). Delivery of TALENs or reTALENs targeting the insertion sequence, together with a promoter-less GFP donor construct, leads to DSB-induced HDR repair of the GFP cassette so that GFP repair efficiency can be used to evaluate the nuclease cutting efficiency (34). We found that reTALENs induced GFP repair in 1.4% of the transfected cells, similar to that achieved by TALENs (1.2%) (Figure 1b). We further tested the activity of reTALENs at the AAVS1 locus in PGP1 hiPSCs (Figure 1c) and successfully recovered cell clones containing specific insertions (Figure 1d and e), confirming that reTALENs are active in both somatic and pluripotent human cells.

We then confirmed that the elimination of repeats would enable us to generate functional lentivirus with a re-TALE cargo. Specifically, we packaged lentiviral particles encoding re-TALE-2 A-GFP and obtained lentiviral particles with tittering of 1.3 × 10^6^ We then tested the activity of the re-TALE-TF encoded by viral particles by transfecting a mCherry reporter into a pool of lenti-reTALE-2 A-GFP-infected 293 T cells. The 293 T cells transduced by lenti-re-TALE-TF showed 36× reporter expression activation compared with the reporter only negative (Supplementary Figure S3a–c). We further checked the sequence integrity of the re-TALE-TF in the lentiviral infected cells and detected full-length reTALEs in all 10 of the clones tested (Supplementary Figure S3d).

### Comparison of ReTALEs and Cas9-gRNA efficiency in hiPSCs with GEAS

**Figure 2.**
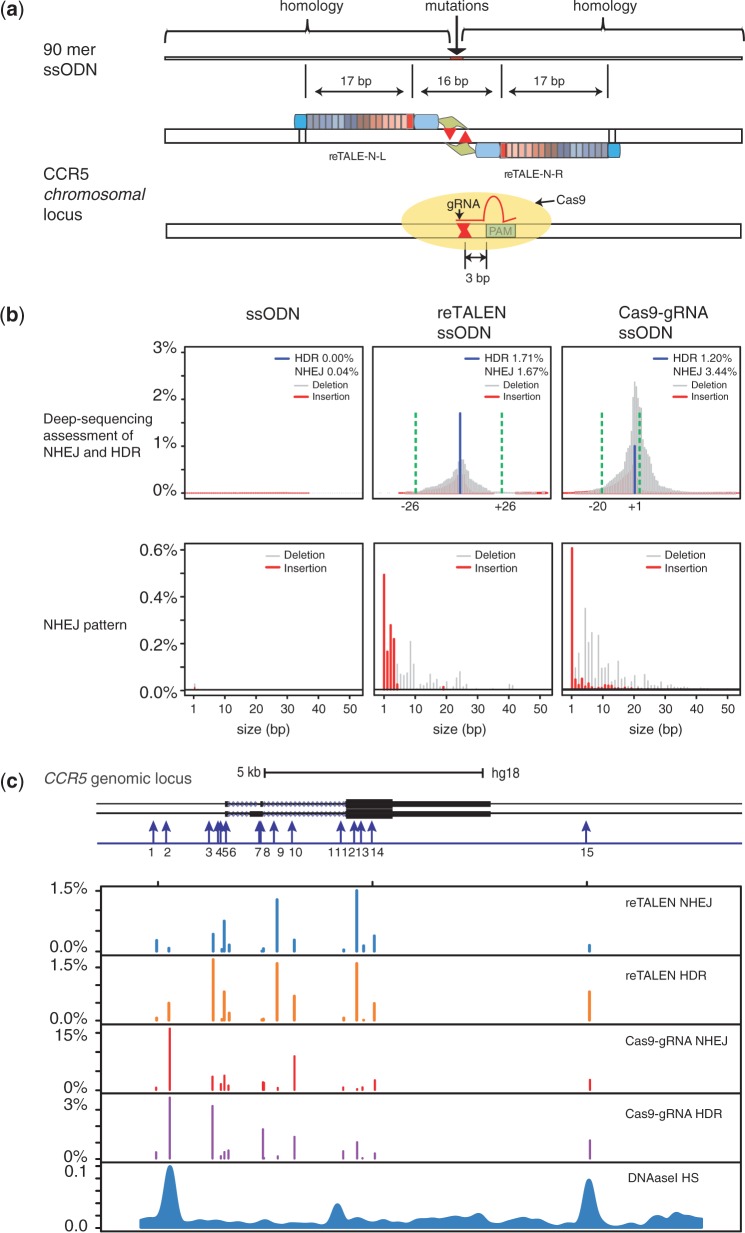
Comparison of reTALENs and Cas9-gRNAs genome targeting efficiency on *CCR5* in iPSCs. (**a**) Schematic representation of genome engineering experimental design. At the re-TALEN pair or Cas9-gRNA targeting site, a 90 mer ssODN carrying a 2 bp mismatch against the genomic DNA was delivered along with the reTALEN or Cas9-gRNA constructs into PGP1 hiPSCs. The cutting sites of the nucleases are depicted as red arrows in the figure. (**b**) Deep-sequencing analysis of HDR and NHEJ efficiencies for re-TALEN pairs (CCR5 #3) and ssODN, or the Cas9-gRNA and ssODN. Alterations in the genome of hiPSCs were analyzed from high-throughput sequence data by GEAS. Top: HDR was quantified from the fraction of reads that contained a 2 bp point mutation built into the center of the ssODN (blue), and NHEJ activity was quantified from the fraction of deletions (gray)/Insertions (red) at each specific position in the genome. For the reTALEN and ssODN graphs, we plot green dashed lines to mark the outer boundary of the re-TALEN pair’s binding sites, which are at positions −26 bp and +26 bp relative to the center of the two re-TALEN-binding sites. For Cas9-gRNA and ssODN graphs, the green dashed lines mark the outer boundary of the gRNA targeting site, which are at positions −20 and −1 bp relative to the Protospacer Associated Motif sequence. Bottom: Deletion/Insertion size distribution in hiPSCs analyzed from the entire NHEJ population with treatments indicated earlier in the text. (**c**) The genome-editing efficiency of re-TALENs and Cas9-gRNAs targeting *CCR5* in PGP1 hiPSCs. Top: schematic representation of the targeted genome-editing sites in *CCR5*. The 15 targeting sites are illustrated by blue arrows later in the text. For each site, cells were co-transfected with a pair of re-TALENs and their corresponding ssODN donor carrying 2 bp mismatches against the genomic DNA. Genome-editing efficiencies were assayed 6 days after transfection. Similarly, we transfected 15 Cas9-gRNAs with their corresponding ssODNs individually into PGP1-hiPSCs to target the same 15 sites and analyzed the efficiency 6 days after transfection. Bottom: the genome-editing efficiency of re-TALENs and Cas9-gRNAs targeting CCR5 in PGP1 hiPSCs. Panels 1 and 2 indicate NHEJ and HDR efficiencies mediated by reTALENs. Panels 3 and 4 indicate NHEJ and HDR efficiencies mediated by Cas9-gRNAs. NHEJ rates were calculated by the frequency of genomic alleles carrying deletions or insertions at the targeting region; HDR rates were calculated by the frequency of genomic alleles carrying 2 bp mismatches. Panel 5, the DNaseI HS profile of a hiPSC cell line from ENCODE database (Duke DNase HS, iPS NIHi7 DS). Of note, the scales of different panels are different.

To compare the editing efficiencies of re-TALENs versus Cas9-gRNA in hiPSCs, we developed a next-generation sequencing platform to precisely pinpoint and quantify both NHEJ and HDR gene-editing events, which we refer to as Genome Editing Assessment System (GEAS). First, we designed and constructed a re-TALEN pair and a Cas9-gRNA, both targeting the upstream region of CCR5 (re-TALEN, Cas9-gRNA pair #3 in Supplementary Table S3), along with a 90 nt ssODN donor identical to the target site except for a 2 bp mismatch (Figure 2a). We then transfected the nuclease constructs and donor ssODN into hiPSCs. To precisely quantitate the gene-editing efficiency, we conducted paired-end deep sequencing on the target genomic region 3 days after transfection. HDR efficiency was measured by the percentage of reads containing the precise 2 bp mismatch. NHEJ efficiency was measured by the percentage of reads carrying indels.

Delivery of the ssODN alone into hiPSCs resulted in minimal HDR and NHEJ rates, whereas delivery of the re-TALENs and the ssODN led to efficiencies of 1.7% HDR and 1.2% NHEJ (Figure 2b). The introduction of the Cas9-gRNA with the ssODN led to 1.2% HDR and 3.4% NHEJ efficiencies. Notably, the rate of genomic deletions and insertions peaked in the middle of the spacer region between the two reTALENs binding site, but peaked 3–4 bp upstream of the protospacer associated motif (PAM) sequence of Cas9-gRNA-targeting site (Figure 2b) as would be expected from the fact that DSBs take place in these regions. We observed a median genomic deletion size of 6 bp and insertion size of 3 bp generated by the re-TALENs and a median deletion size of 7 bp and insertion of 1 bp by the Cas9-gRNA (Figure 2b), consistent with DNA lesion patterns usually generated by NHEJ (4). Several analyses of our next-generation sequencing platform revealed that GEAS can detect HDR detection rates as low as 0.007%, which is both highly reproducible (coefficient of variation between replicates = ± 15% × measured efficiency) and 400× more sensitive than most commonly used mismatch sensitive endonuclease assays (Supplementary Figure S4).

After confirming the reliability of GEAS, we next sought to test the scalability of our tools by building and assessing re-TALEN pairs and Cas9-gRNAs targeted to 15 sites at the CCR5 genomic locus (Figure 2c, Supplementary Table S3). Anticipating that editing efficiency might depend on chromatin state, these sites were selected to represent a wide range of DNaseI sensitivities (35). The nuclease constructs were transfected with the corresponding ssODNs donors (Supplementary Table S3) into PGP1 hiPSCs. Six days after transfection, we profiled the genome-editing efficiencies at these sites (Supplementary Table S4). For 13 of 15 re-TALEN pairs with ssODN donors, we detected NHEJ and HDR at levels above our statistical detection thresholds, with an average NHEJ efficiency of 0.4% and an average HDR efficiency of 0.6% (Figure 2c). In addition, a statistically significant positive correlation (*r*^2 ^= 0.81) was found between HR and NHEJ efficiency at the same targeting loci (*P* < 1 × 10^−^^4^) (Supplementary Figure S5a), suggesting that DSB generation, the common upstream step of both HDR and NHEJ, is a rate-limiting step for reTALEN-mediated genome editing.

In contrast, all 15 Cas9-gRNA pairs showed significant levels of NHEJ and HR, with an average NHEJ efficiency of 3% and an average HDR efficiency of 1.0% (Figure 2c). In addition, a positive correlation was also detected between the NHEJ and HDR efficiency introduced by Cas9-gRNA (Supplementary Figure S5b) (*r*^2 ^= 0.52, *P* = 0.003), consistent with what we had observed with our reTALENs. The NHEJ efficiency achieved by Cas9-gRNA was significantly higher than that achieved by reTALENs (*t*-test, paired-end, *P* = 0.02). Interestingly, we observed a moderate but statistically significant correlation between NHEJ efficiency and the melting temperature of the gRNA targeting sequence (Supplementary Figure S5c) (*r*^2 ^= 0.28, *P* = 0.04), suggesting that the strength of base pairing between the gRNA and its genomic target could explain as much as 28% of the variation in the efficiency of Cas9-gRNA-mediated DSB generation. Even though Cas9-gRNA produced NHEJ levels at an average of seven times higher than the corresponding reTALEN, Cas9-gRNA only achieved HDR levels (average = 1.0%) similar to that of the corresponding reTALENs (average = 0.6%), suggesting either that the ssODN concentration at the DSB is the limiting factor for HDR or that the genomic break structure created by the Cas9-gRNA is not favorable for effective HDR (see ‘Discussion’ section). Of note, within our data, we did not observe any correlation between DNaseI HS and the genome targeting efficiencies achieved by either method (Supplementary Figure S6).

### Optimization of ssODN donor design for HDR

Although ssODNs have been found to be effective as donor DNA in genome editing [see earlier in the text, (21,22)], many questions remain regarding how to optimize their design. Having compared the efficiencies of reTALEN and Cas9-gRNA nucleases, we next developed strategies for the design of highly performing ssODNs in hiPSCs.

**Figure 3.**
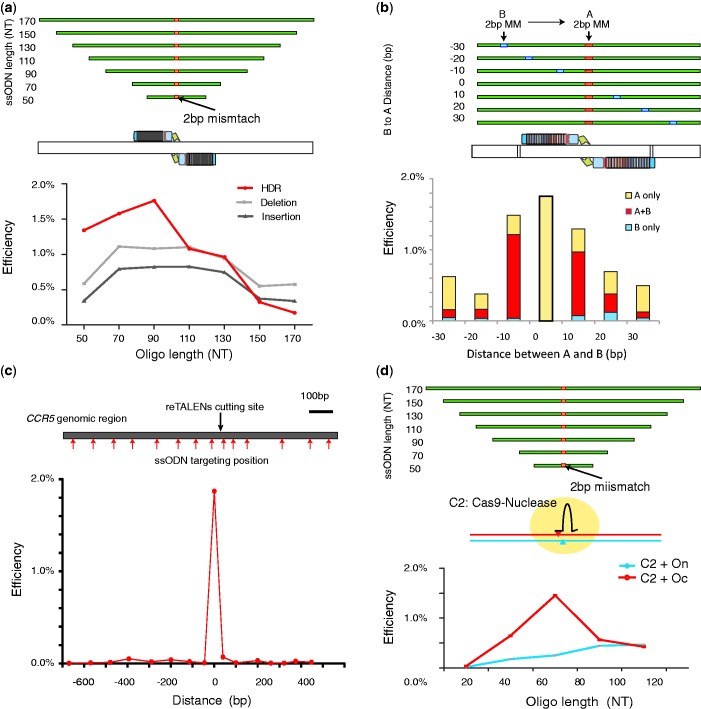
Study of functional parameters governing ssODN-mediated HDR with re-TALENs or Cas9-gRNAs in PGP1 hiPSCs. (**a**) PGP1 hiPSCs were co-transfected with re-TALENs pair (#3) and ssODNs of different lengths (50, 70, 90, 110, 130, 150 and 170 nt). All ssODNs possessed an identical 2 bp mismatch against the genomic DNA in the middle of their sequence. A 90 mer ssODN achieved optimal HDR in the targeted genome. The assessment of HDR, NHEJ-incurred deletion and insertion efficiency is described in the ‘Materials and Methods’ section. (**b**) 90 mer ssODNs corresponding to re-TALEN pair #3 each containing a 2 bp mismatch (A) in the center and an additional 2 bp mismatch (B) at different positions offset from A (where offsets varied from −30 to 30 bp) were used to test the effects of deviations from homology along the ssODN. Genome-editing efficiency of each ssODN was assessed in PGP1 hiPSCs. The bottom bar graph shows the incorporation frequency of A only, B only and A + B in the targeted genome. HDR rates decrease as the distance of homology deviations from the center increase (see text and Supplementary Figure S7a and b). (**c**) ssODNs targeted to sites with varying distances (−620∼480 bp) away from the target site of re-TALEN pair #3 were tested to assess the maximum distance within which we can place ssODNs to introduce mutations. All ssODNs carried a 2 bp mismatch in the middle of their sequences. We observed minimal HDR efficiency (≤0.06%) when the ssODN mismatch was positioned 40 bp away from the middle of re-TALEN pair’s binding site. (**d**) PGP1 hiPSCs were co-transfected with Cas9-gRNA (AAVS1) and ssODNs of different orientation (O_c_: complement to gRNA; O_n_: non-complement to gRNA) and different lengths (30, 50, 70, 90 and 110 nt). All ssODNs possessed an identical 2 bp mismatch against the genomic DNA in the middle of their sequence. A 70 mer O_c_ achieved optimal HDR in the targeted genome.

We first designed a set of ssODNs donors of different lengths (50–170 nt), all carrying the same 2 bp mismatch in the middle of the spacer region of the CCR5 re-TALEN pair #3 target sites. HDR efficiency was observed to vary with ssODN length, and an optimal HDR efficiency of ∼1.8% was observed with a 90 nt ssODN, whereas longer ssODNs decreased HDR efficiency (Figure 3a). As longer homology regions improve HDR rates when dsDNA donors are used with nucleases (36), possible reasons for this result may be that ssODNs are used in an alternative genome repair process; longer ssODNs are less available to the genome repair apparatus or that longer ssODNs incur negative effects that offset any improvements gained by longer homology, compared with dsDNA donors (37). Yet, if either of the first two reasons were the case, then NHEJ rates should either be unaffected or would increase with longer ssODNs because NHEJ repair does not involve the ssODN donor. However, NHEJ rates were observed to decline along with HDR (Figure 3a), suggesting that the longer ssODNs present offsetting effects. Possible hypotheses would be that longer ssODNs are toxic to the cell (38) or that transfection of longer ssODNs saturates the DNA processing machinery, thereby causing decreased molar DNA uptake and reducing the capacity of the cells to take up or express re-TALEN plasmids.

Next, we examined how rate of incorporation of a mismatch carried by the ssODN donor varies with its distance to the DSB. To this end, we designed a series of 90 nt ssODNs all possessing the same 2 bp mismatch (A) in the center of the spacer region of re-TALEN pair #3. Each ssODN also contained a second 2 bp mismatch (B) at varying distances from the center (Figure 3b). An ssODN possessing only the center 2 bp mismatch was used as a control. Each of these ssODNs was introduced individually with re-TALEN pair #3, and the outcomes were analyzed with GEAS. We found that overall HDR—as measured by the rate at which the A mismatch was incorporated (A only or A + B)—decreased as the B mismatches became farther from the center (Figure 3b, Supplementary Figure S7a). The higher overall HDR rate observed when B is only 10 bp away from A may reflect a lesser need for annealing of the ssODN against genomic DNA immediately proximal to the dsDNA break.

For each distance of B from A, a fraction of HDR events only incorporated the A mismatch, whereas another fraction incorporated both A and B mismatches [Figure 3b (A only and A + B)], These two outcomes may be due to gene conversion tracts (39) along the length of the ssDNA oligo, whereby incoporation of A + B mismatches resulted from long conversion tracts that extended beyond the B mismatch, and incorporation of the A-only mismatch resulted from shorter tracts that did not reach B. Under this interpretation, we estimated a distribution of gene conversion lengths in both directions along the ssODN (Supplementary Figure S7b). The estimated distribution implies that gene conversion tracts progressively become less frequent as their lengths increase, a result similar to gene conversion tract distributions seen with dsDNA donors (39), but on a highly compressed distance scale of tens of bases for the ssDNA donor versus hundreds of bases for dsDNA donors. Consistent with this result, an experiment with a ssODN containing three pairs of 2 bp mismatches spaced at intervals of 10 nt on either side of the central 2 bp mismatch ‘A’s gave rise to a pattern in which A alone was incorporated 86% of the time, with multiple B mismatches incorporated at other times (Supplementary Figure S7c). Although the numbers of B only incorporation events were too low to estimate a distribution of tract lengths <10 bp, it is clear that the short tract region within 10 bp of the nuclease site predominates (Supplementary Figure S7b). Finally, in all of our experiments with single B mismatches, we see a small fraction of B-only incorporation events (0.04–0.12%) that is roughly constant across all B distances from A. The nature of these events is unclear.

Furthermore, we tested how far the ssODN donor can be placed from the re-TALEN-induced dsDNA break and still observe incorporation. A set of 90 nt ssODNs with central 2 bp mismatches targeting a range of larger distances (−600 to +400 bp) away from the re-TALEN-induced dsDNA break site were tested. When the ssODNs matched ≥40 bp away, we observed >30× lower HDR efficiencies compared with the control ssODN positioned centrally over the cut region (Figure 3c). The low level of incorporation that was observed may be due to processes unrelated to the dsDNA cut, as seen in experiments in which genomes are altered by a ssDNA donor alone (38). Meanwhile, the low level of HDR present when the ssODN is ∼40 bp away may be due to a combination of weakened homology on the mismatch-containing side of the dsDNA cut along with insufficient ssODN oligo length on the other side of the dsDNA break.

We similarly tested the ssODNs DNA donor design for Cas9-gRNA-mediated targeting. First, we constructed Cas9-gRNA (C_2_) targeting the AAVS1 locus and designed ssODN donors of variable orientations (O_c_: complementary to the gRNA and O_n_: non-complementary to the gRNA) and lengths (30, 50, 70, 90 and 110 nt). We found O_c_ achieved better efficiency than O_n_, with a 70 mer O_c_ achieving an optimal HDR rate of 1.5%. (Figure 3d) The same ssODN strand bias was detected using a Cas9-derived nickase (C_c_: Cas9_D10A), despite the fact that the HDR efficiencies mediated by C_c_ with ssODN were significantly less than C_2_ (*t*-test, paired-end, *P* = 0.02) (Supplementary Figure S8). Future investigation will further elucidate the factors that may contribute to this bias, including sequence bias, direction of transcription and replication.

### hiPSC clonal isolation of corrected cells

GEAS revealed that re-TALEN pair #3 achieved precise genome editing with an efficiency of ∼1% in hiPSCs, a level at which correctly edited cells can usually be isolated by screening clones. HiPSCs have poor viability as single cells, but recent advances in culture conditions have facilitated outgrowth of hiPSCs from single cells (23). We optimized these protocols along with a single-cell FACS sorting procedure to establish a robust platform for single hiPSCs sorting and maintenance, where hiPSC clones can be recovered with survival rates of >25% (see ‘Materials and Methods’ section). We combined this method with a rapid and efficient genotyping system where we can conduct chromosomal DNA extraction and targeted genome amplification in 1-h single tube reactions, enabling large-scale genotyping of edited hiPSCs. Together, these methods comprise a pipeline for robustly obtaining genome-edited hiPSCs without selection.

**Figure 4.**
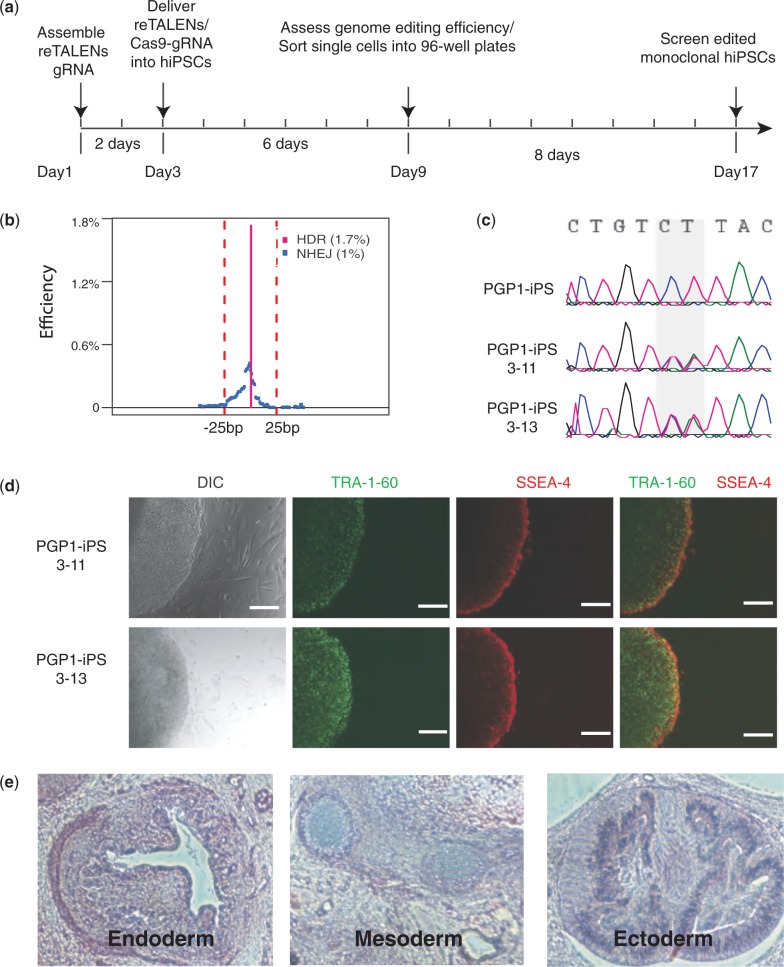
Using re-TALENs and ssODNs to obtain monoclonal genome-edited hiPSC without selection. (**a**) Timeline of the experiment. (**b**) Genome engineering efficiency of re-TALENs pair and ssODN (#3) assessed by the NGS platform described in Figure 2b. (**c**) Sanger sequencing results of monoclonal hiPSC colonies after genome editing. Of note, the 2 bp heterogeneous genotype (CT/CT→TA/CT) was successfully introduced into the genome of PGP1-iPS-3-11, PGP1-iPS-3-13 colonies. (**d**) Immunofluorescence staining of targeted PGP1-iPS-3-11. Cells were stained for the pluripotency markers Tra-1-60 and SSEA4. (**e**) Hematoxylin and eosin staining of teratoma sections generated from monoclonal PGP1-iPS-3-11 cells.

To demonstrate this system (Figure 4a), we first transfected PGP1 hiPSCs with a pair of re-TALENs and an ssODN targeting CCR5 at site #3 (Supplementary Table S3), and we performed GEAS with a portion of the transfected cells, finding an HDR frequency of 1.7% (Figure 4b). This information, along with the 25% recovery of sorted single-cell clones, allowed us to estimate that we could obtain at least one correctly edited clone from five 96-well plates with Poisson probability 98% (assuming µ = 0.017 × 0.25 × 96 × 5 × 2). Six days after transfection, hiPSCs were FACS sorted and 8 days after sorting, 100 hiPSC clones were screened. Sanger sequencing revealed that 2 of 100 of these unselected hiPSC colonies contained a heterozygous genotype possessing the 2 bp mutation introduced by the ssODN donor (Figure 4c). The targeting efficiency of 1% (1% = 2/2 × 100, 2 mono-allelic corrected clones out of 100 cell screened) was consistent with the next-generation sequencing analysis (1.7%) (Figure 4b). The pluripotency of the resulting hiPSCs was confirmed with immunostaining for SSEA4 and TRA-1-60 (Figure 4d). The successfully targeted hiPSCs clones were able to generate mature teratomas with features of all three germ layers (Figure 4e).

## DISCUSSION

Here, we developed and demonstrated several improvements to the design and assessment of genome-editing reagents and demonstrated a streamlined method for efficient human stem cell editing. We first developed reTALENs, which simplify TALEN construction and enables the generation of functional lenti-viruses, which are important tools for delivering the reagents into many cell types and animals (33).

We then built a highly sensitive GEAS assay system to easily and precisely pinpoint and quantify HDR and NHEJ events in hiPSCs. In comparison with other methods of assessing design parameters for genome-editing, our genome-editing assessment tool provides simultaneous information on rates of HDR, NHEJ and other mutagenic processes through a single experimental and statistical analysis method versus performing different experiments and applying separate statistical methods for each individually. In the course of this study, we routinely pooled ∼50 barcoded samples together and used the Illumina MiSeq system to obtain the sequence data, which was analyzed with our genome-editing assessment software. Currently, MiSeq can deliver ∼20 Million paired-end 150 bp reads within 27 h so that up to 200 sample-barcoded targeting regions can be covered with ∼100 K reads each at a cost of approximately $5 per sample. If desired, sample throughput can be traded off for higher sensitivity by allotting more reads per sample and processing fewer samples. Software and documentation for our genome-editing assessment system is available to provide researchers with the means to improve and standardize their genome-editing methods and extend them to additional cell lines and types.

Using our developed reTALENs, Cas9-gRNAs and GEAS method, we compared HDR and NHEJ efficiencies across 15 pairs of reTALENs and Cas9-gRNA (Supplementary Table S3 and S4) on the CCR5 locus. We found 13/15 of reTALEN pairs and all 15 Cas9-gRNAs exhibited detectable activities in hiPSCs, suggesting that both nuclease platforms serve as robust tools for genome editing. We confirmed the activity of the two failed reTALEN pairs in K562 cells and found 4 and 3% cutting efficiency, respectively, suggesting some pertinent factors in hiPSCs, such as heterochromatin of methylation at the targeting regions make them resistant to reTALEN activity. In addition, we found that Cas9-gRNA induced on average 7–8× greater NHEJ rates than reTALEN, similar to recent reports (15). The effective concentration of Cas9-gRNA complexes or the intrinsic enzyme kinetics may contribute to this difference. Surprisingly, we did not see an equivalent increase of HDR with Cas9-gRNA and ssODN. Although ssODN concentration may reach saturating levels during construct delivery, ssODN availability at the DSB might be the limiting factor for HDR. Future studies using Cas9-gRNA nickases to generate defined DSB resections more favorable for HDR (36) can be conducted to test this hypothesis and further increase HDR efficiencies. Although we have compared the genome-targeting efficiencies achieved by reTALENs and Cas9-gRNA, a critical issue will also be to determine the generation of off-target mutations. It will be imperative to address the specificity of both targeting tools to improve the potential of hiPSCs genome engineering.

Finally, we demonstrated a streamlined pipeline for obtaining scarlessly edited human stem cells using our reagents. The pipeline comprises of the following: (i) reTALEN or Cas9-gRNA synthesis; (ii) prospective screening of reagents using GEAS; and (iii) high-throughput isolation of hiPSC clones. We note that with 1% HDR efficiency, it is feasible to generate isogeneic hiPSCs with mono-allelic mutations, which will facilitate hiPSC-based modeling of dominant alleles, allele-specific expression or X-linked mutations. However, targeting efficiencies must be improved to generate of homozygous mutations in hiPSCs. Other strategies such as transfection enrichment (15,17), or transient hypothermia (40), can be used together with our tools to achieve this goal. Last, we emphasize the versatility of our tools in that re-TALEs/Cas-gRNA can be engineered and used for other genomic-targeting technologies such as customized transcriptional factors and epigenetic modifiers, whereas GEAS can be applied to other gene-editing techniques, such as ZFNs, targeted nickases and meganucleases. We envision that our pipeline of efficiently generating scarlessly engineered human stem cells will allow the research community to resolve the causal underpinnings of numerous important biological problems, as well as to precisely engineer hiPSCs and other cell lines for autologous cell therapy.

## SUPPLEMENTARY DATA

Supplementary Data are available at NAR Online.

## FUNDING

National Human Genome Research Institute (NHGRI) Center for Excellence in Genomics Science [P50 HG005550, G.M.C.]; funded by Human Frontiers Science Program long-term fellowship (to M.G.). Funding for open access charge: NHGRI Center for Excellence in Genomics Science [P50 HG005550, G.M.C.].

*Conflict of interest statement*. G.M.C., L.Y, M.G. and J.Y. are inventors on a patent application describing the reTALE concept and assembly method.

## Supplementary Material

Supplementary Data
