# Supplementary material for: Optimization of scarless human stem cell genome editing
Source: Nucleic Acids Res. 2013 Jul 31;41(19):9049–61. doi: 10.1093/nar/gkt555 (PMC3799423; doi:10.1093/nar/gkt555)
Supplement: Supplementary Data [file supp_41_19_9049__index.html]

Optimization of scarless human stem cell genome editing — Optimization of scarless human stem cell genome editing — Supplementary Data 

# Optimization of scarless human stem cell genome editing

## 

files

**Files in this Data Supplement:**

- Supplementary Data - pdf file
