# Supplementary material for: Optimization of scarless human stem cell genome editing
Source: Nucleic Acids Res. 2013 Jul 31;41(19):9049–61. doi: 10.1093/nar/gkt555 (PMC3799423; doi:10.1093/nar/gkt555)

Supplementary Information

Optimization of Scarless Human Stem Cell Genome Editing

Luhan Yang[1,2], Marc Guell[1], Susan Byrne[1,6], Joyce Yang[1,2,6], Alejandro De Los Angeles[3,6], Prashant Mali[1], John Aach[1] , Adrian W Briggs[1], Xavier Rios[1], Po-Yi Huang[1,4], George Daley[3], and George Church[1,5] *

[1]Department of Genetics, Harvard Medical School, Boston, Massachusetts, USA

[2]Biological and Biomedical Sciences Program, Harvard Medical School, Boston, Massachusetts, USA

[3]Children's Hospital, Boston, Massachusetts, USA

[4]Chemistry and Chemical Biology program, Harvard, Cambridge, Massachusetts, USA

[5]Wyss Institute for Biologically Inspired Engineering, Harvard University, Cambridge,

[6]These authors contributed equally to this work

*Correspondence: gchurch@genetics.med.harvard.edu.

Index	Content
Supplementary Note 1	Assembly strategy of gRNA and reTALE
Supplementary Note 2	Statistical analysis of genome editing NGS data
Supplementary Figure 1	Design of reTALE
Supplementary Figure 2	Design and practice of TALE Single-incubation Assembly (TASA) assembly
Supplementary Figure 3	The functionality and sequence integrity of Lenti-reTALEs
Supplementary Figure 4	The sensitivity and reproducibility of GEAS
Supplementary Figure 5	Statistical analysis of NHEJ and HDR efficiencies by reTALENs and Cas9-gRNAs on *CCR5.*
Supplementary Figure 6	The correlation analysis of genome editing efficiency and epigenetic state.
Supplementary Figure 7	The impact of homology pairing in the ssODN-mediated genome editing.
Supplementary Figure 8	Cas9-gRNA nuclease and nickases genome editing efficiencies
Supplementary Figure 9	The design and optimization of re-TALE sequence

supplementary Table 1	re-TALE blocks sequences
supplementary Table 2	re-TALE blocks primer sequences
Supplementary Table3	re-TALEN pairs and Cas9-gRNAs targeting *CCR5*
Supplementary Table4	HDR and NHEJ efficiency of re-TALENs and Cas9-gRNAs targeting *CCR5*
Supplementary Table5	CCR5 targeting site PCR primer sequences
Supplementary Table6	ssODN design for studying ssODN-mediated genome editing
Supplementary Sequence 1	re-TALE sequence
Supplementary Sequence 2	re-TALEN and re-TALE-TF backbone sequence
Supplementary Sequence 3	gRNA backbone sequence

Supplementary Note1: Assembly strategy of gRNA and reTALE

We first devised a robust protocol in which gRNA for CAS9-mediated genome editing can be synthesized directly by incubating two 100mer oligos of customized sequence with the linearized backbone in an isothermal assembly mixture. We detected >90% assembly efficiency as confirmed by Sanger sequencing. In parallel, to expedite reTALE construct synthesis, we created a library of RVD dimer blocks and backbone constructs (Supplementary Figure 2a) for a robust and cost-effective assembly protocol (TASA, TALE Single-incubation Assembly). TASA enabled us to assemble re-TALEs in a one pot one hour reaction (Supplement Figure. 2b). We found perfect re-TALE assemblies with the following success rates: re-TALE-12.5, 46%; re-TALE-14.5, 32%; and re-TALE16.5, 18% (Supplement Fig. 2C). Alternatively, re-TALE16.5s can be assembled in a two-stage protocol (Material and Methods) with 90% efficiency.

Supplementary Note2: Statistical analysis of genome editing NGS data

(1) HDR specificity analysis

We used an exact binomial test to compute the probabilities of observing various numbers of sequence reads containing the 2bp mismatch. Based on the sequencing results of 10bp windows before and after the targeting site, we estimated the maximum base change rates of the two windows (P1 and P2). Using the null hypothesis that the changes of each of the two target bp were independent, we computed the expected probability of observing 2bp mismatch at the targeting site by chance as the product of these two probabilities (P1*P2). Given a dataset

containing N numbers of total reads and n number of HDR reads, we calculated the p-value of the observed HDR efficiency.

(2) HDR sensitivity analysis

In our experimental design, the ssODN DNA donors contained a 2bp mismatch against the targeting genome, so that we expected co-presence of the base changes in the two target bp if the ssODN was incorporated into the targeting genome. Other non-intended observed sequence changes would not likely change at the same time. Thus, we predicted non-intended changes to be much less interdependent. Based on these assumptions, we used mutual information (MI) to measure the mutual dependence of simultaneous two base pair changes in all other pairs of positions, and we estimated the HDR detection limit as the smallest HDR where MI of the targeting 2bp site is higher than MI of all the other position pairs. For a given experiment, we first identified HDR reads with intended 2bp mismatch from the original fastq file and we simulated a set of fastq files with diluted HDR efficiencies by systematically removing different numbers of HDR reads from the original data set. Mutual information (MI) was computed between all pairs of positions within a 20bp window centered on the targeting site. In these calculations, the mutual information of the base composition between any two positions is computed. Thus, unlike our HDR specificity measure above, this measure does not assess the tendency of position pairs to change to any particular pairs of target bases, only their tendency to change at the same time. (Figure S4A, Table S4). We coded our analysis in R and MI was computed using the package infotheo.

(3) Correlations between genome editing efficiency and epigenetic state

We computed Pearson correlation coefficients to study possible associations between epigenetic parameters (DNase I HS or nucleosome occupancy) and genome engineering efficiencies (HDR, NHEJ). Dataset of DNAaseI Hypersensitivity was downloaded from UCSC genome browser.

hiPSCs DNase I HS: /gbdb/hg19/bbi/wgEncodeOpenChromDnaseIpsnihi7Sig.bigWig

To compute P-values, we compared the observed correlation to a simulated distribution which was built by randomizing the position of the epigenetic parameter (N=100000). Observed correlations higher than the 95th percentile, or lower than the 5th percentile of the simulated distribution were considered as potential associations.

Supplementary Figure 1. Design of reTALE. (a) Sequence alignment of the original TALE RVD monomer with monomers in re-TALE-16.5 (re-TALE-M1→re-TALE-M17). Nucleotide alterations

from the original sequence are highlighted in gray. (b) Test of repetitiveness of re-TALE by PCR. Top panel illustrates the structure of re-TALE/TALE and positions of the primers in the PCR reaction. Bottom panel illustrates PCR bands with condition indicated below. Note the PCR laddering presents with the original TALE template (right lane).

Supplementary Figure 2. Design and practice of TALE Single-incubation Assembly (TASA) assembly.

(a) Schematic representation of the library of re-TALE dimer blocks for TASA assembly. There is a library of 10 re-TALE dimer blocks encoding two RVDs. Within each block, all 16 dimers share the same DNA sequence except the RVD encoding sequences; Dimers in different blocks have distinct sequences but are designed such that they share 32bp overlaps with the adjacent blocks. DNA and amino acid sequence of one dimer (Block6_AC) are listed on the right.

(b) Schematic representation of TASA assembly. The left panel illustrates the TASA assembly method: a one-pot incubation reaction is conducted with an enzyme mixture/re-TALE blocks/re-TALE-N/TF backbone vectors. The reaction product can be used directly for bacterial transformation. The right panel illustrates the mechanism of TASA. The destination vector is linearized by an endonuclease at 37°C to cut off *ccd*B counter-selection cassette; the exonuclease, which processes the end of blocks and linearized vectors, exposes ssDNA overhangs at the end of fragments to allow blocks and vector backbones to anneal in a designated order. When the temperature rises up to 50°C, polymerases and ligases work together to seal the gap, producing the final constructs ready for transformation.

(c) TASA assembly efficiency for re-TALEs possessing different monomer lengths. The blocks used for assembly are illustrated on the left and the assembly efficiency is presented on the right.

Supplementary Figure 3. The functionality and sequence integrity of Lenti-reTALEs.

(a) Schematic representation of the fluorescence reporter system for testing the activity lentiviral particle encoding re-TALE. The diagram illustrates the structure of re-TALE-TF-2A-GFP constructs and its mCherry reporters. VP64, synthetic transcription activation domain; 2A, self-cleavage peptides.

(b) Tittering lenti-reTALE-TF-2A-GFP stock. We infected fresh 293T cells with different volume of lentivirus-containing suspension, and measured GFP positive cells 3 days after transduction. We determined tittering is $1.3*10^6$ Transduction Unite/ml.

(c) Test of the lentiviral reTALE activity: Top: Images of lentivirus-transduced 293T cells transfected with mCherry reporter plasmid. We transduced $5*10^5$ 293T cells with lentiviral particles encoding re-TALE-TF-2A-GFP using 100μl of lentiviral suspension. Three days after transduction, we transfected the transduced cells with the corresponding 30ng mCherry reporter

to verify the activity of lenti-TALE-TF/lenti-re-TALE-TF. Scale bar, 100 μm. Bottom: Representative FACS plot measuring the GFP and mCherry signal of lentivirus-transduced 293T cells transfected with mCherry reporter plasmid. The expression activation fold was calculated by the ratio of mCherry signal strength with vs. without activation (mean mCherry Q2/mean mCherry Q3).

(d) PCR of genomic DNA of 10 independent colonies infected by lentiviral particles encoding re-TALE-TF. We found all the colonies carried desired full length reTALE cassette.

Supplementary Figure 4. The sensitivity and reproducibility of GEAS

(A) Information-based analysis of HDR detection limit. Given the dataset of re-TALENs (#10)/ssODN, we identified the reads containing the expected editing (HDR) and systematically removed these HDR reads to generate different artificial datasets with a "diluted" editing signal. We generated datasets with 100, 99.8, 99.9, 98.9, 97.8, 89.2, 78.4, 64.9, 21.6, 10.8, 2.2, 1.1, 0.2, 0.1, 0.02, and 0% removal of HDR reads to generate artificial datasets with HR efficiency ranging from 0~0.67%. For each individual dataset, we estimated mutual information (MI) of the background signal (in purple) and the signal obtained in the targeting site (in green). We observe that MI at the targeting site is remarkably higher than the background when the HDR efficiency is above 0.0014%. We estimated a limit of HDR detection between 0.0014% and 0.0071%. MI calculation is described in the Methods.

(B) The test of reproducibility of genome editing assessment system. The pairs of plots (Top and Bottom) show the HDR and NHEJ assessment results of two replicates with re-TALENs pair and cell type indicated above. For each experiment, we conducted nucleofection, targeted genome amplification, deep-sequencing and data analysis independently. We calculated the genome editing assessment variation of replicates as √2 (|HDR1-HDR2|)/((HDR+HDR2)/2) =ΔHDR/HDR and √2 (|NHEJ1-NHEJ2|) /((NHEJ1+NHEJ2)/2) =ΔNHEJ/NHEJ and listed the variation results below the plots. We calculated the average variation of our system by (19%+11%+4%+9%+10%+35%)/6=15%. Factors that may contribute to the variations include the status of cells under nucleofection, nucleofection efficiency, and sequencing coverage and quality.

Supplementary Figure 5. Statistical analysis of NHEJ and HDR efficiencies by reTALENs and Cas9-gRNAs on *CCR5*.

(a) The correlation of HR and NHEJ efficiencies mediated by reTALENs at identical sites in iPSCs (r=0.91, P< 1X 10^{-5}).

(b) The correlation of HR and NHEJ efficiencies mediated by Cas9-gRNA at identical sites in iPSCs (r=0.74, P=0.002).

(c) The correlation of NHEJ efficiencies mediated by Cas9-gRNA and the Tm temperature of gRNA targeting site in iPSCs (r=0.52, P=0.04)

Supplementary Figure 6. The correlation analysis of genome editing efficiency and epigenetic state.

We used Pearson correlation to study possible associations between DNase I sensitivity and genome engineering efficiencies (HR, NHEJ). We compared the observed correlation to a randomized set (N=100000). Observed correlations higher than the 95th percentile, or lower than the 5th percentile of the simulated distribution were considered as potential associations. We did not observe any significant correlation between DNase1 sensitivity and NHEJ/HR efficiencies.

Supplementary Figure 7. The impact of homology pairing in the ssODN-mediated genome editing.

(a) In the experiment described in Figure 3b, we found that overall HDR as measured by the rate at which the middle 2b mismatch (A) was incorporated decreased as the secondary mismatches B increased their distance from the A (relative position of B to A varies from -30→30bp). The higher rates of incorporation when B is only 10bp away from A (-10bp and +10b) may reflect a lesser need for pairing of the ssODN against genomic DNA proximal to the dsDNA break.

(b) Distribution of gene conversion lengths along the ssODN. We observed that at each distance of B from A, a fraction of HDR events incorporates only A while another fraction incorporates both A and B (see Figure 3b). These two events may be interpretable in terms of gene conversion tracts (Elliott et al., 1998), whereby A+B events represent long conversion tracts that extend beyond B and A-only events represent shorter ones that do not reach to B. Under this interpretation, a distribution of gene conversion lengths in both directions along the oligo can be estimated (we defined the middle of ssODN as 0, conversion tracks towards the 5' end of ssODN as - direction, and 3' end as + direction). Gene conversion tracts progressively decrease in incidence as their lengths increase, a result very similar to gene conversion tract distributions seen with dsDNA donors, but on a highly compressed distance scale of tens of bp for the ssDNA oligo vs. hundreds of bases for dsDNA donors.

(c) Assays for gene conversion tracts using a single ssODN that contains a series of mutations and measuring contiguous series of incorporations. Here, we used an ssODN donor with three pairs of 2bp mismatches (orange) spaced at intervals of 10nt on either side of the central 2bp mismatch (Top). We only detected few genomic sequencing reads (62) carrying >=1 mismatches defined by ssODN among >300,000 reads sequencing this region. We plotted all these reads in the plot (bottom) and the sequence of the reads was color coded. Orange: defined mismatches; green: wild type sequence. Genome editing with this ssODN gave rise of a pattern in which middle mutation alone was incorporated 85% (53/62) of the time, with multiple B mismatches

incorporated at other times. Although numbers of B incorporation events were too low to estimate a distribution of tract lengths > 10bp, it is clear that the short tract region from -10-10bp predominates.

Supplementary Figure 8. Cas9-gRNA nuclease and nickases genome editing efficiencies

PGP1 iPSCs were co-transfected with combination of nuclease (C_2) (Cas9-gRNA, cleaves two strands) or nickase (C_c) (Cas9D10A-gRNA, cleaves the non complementary strand) and ssODNs of different orientation (Oc and On). All ssODNs possessed an identical 2bp mismatch against the genomic DNA in the middle of their sequence. The assessment of HDR is described in the Methods.

Supplement Figure 9. The design and optimization of re-TALE sequence

The re-TALE sequence was evolved in several design cycles to eliminate repeats. In each cycle, synonymous sequences from each repeat are evaluated. Those with the largest hamming distance to the evolving DNA are selected. The final sequence with cai = 0.59 ΔG= -9.8 kcal/mol. We provide an R package to carry out this general framework for synthetic protein design.

Table S1. re-TALE blocks sequences

block0	CGCAATGCGCTCACGGGAGCACCCCTCAACCTAACCCCTGAACAGGTAGTCGCTATAGCTTCANNNNNNGGGGGCAAGCAAGCACTTGAGACCGTTCAACGACTCCTGCCAGTGCTCTGCCAAGCCCATGGATTGACTCCGGAGCAAGTCGTCGCGATCGCGAGCNNNNNNGGGGGGAAGCAGGCGCTGGAAACTGTTCAGAGACTGCTGCCTGTACTTTGTCAGGCGCATGGTCTC
block1	AGACTGCTGCCTGTACTTTGTCAGGCGCATGGTCTCACCCCCGAACAGGTTGTCGCAATAGCAAGTNNNNNNGGCGGTAAGCAAGCCCTAGAGACTGTGCAACGCCTGCTCCCCGTGCTGTGTCAGGCTCACGGTCTGACACCTGAACAAGTTGTCGCGATAGCCAGTNNNNNNGGGGGAAAACAAGCTCTAGAAACGGTTCAAAGGTTGTTGCCCGTTCTGTGCCAAGCACATGGGTTA
block1'	TGCGCTCACGGGAGCACCCCTCAACCTCACCCCCGAACAGGTTGTCGCAATAGCAAGTNNNNNNGGCGGTAAGCAAGCCCTAGAGACTGTGCAACGCCTGCTCCCCGTGCTGTGTCAGGCTCACGGTCTGACACCTGAACAAGTTGTCGCGATAGCCAGTNNNNNNGGGGGAAAACAAGCTCTAGAAACGGTTCAAAGGTTGTTGCCCGTTCTGTGCCAAGCACATGGGTTA
block2	AGGTTGTTGCCCGTTCTGTGCCAAGCACATGGGTTAACACCCGAACAAGTAGTAGCGATAGCGTCANNNNNNGGGGGTAAACAGGCTTTGGAGACGGTACAGCGGTTATTGCCGGTCCTCTGCCAGGCCCACGGACTTACGCCAGAACAGGTGGTTGCAATTGCCTCCNNNNNNGGCGGGAAACAAGCGTTGGAAACTGTGCAGAGACTCCTTCCTGTTTTGTGTCAAGCCCACGGCTTGACGCCT

block3	AGACTCCTTCCTGTTTTGTGTCAAGCCCACGGCTTGACGCCTGAGCAGGTTGTG GCCATCGCTAGCNNNNNNGGAGGGAAGCAGGCTCTTGAAACCGTACAGCGACTT CTCCCAGTTTTGTGCCAAGCTCACGGGCTAACCCCCGAGCAAGTAGTTGCCATA GCAAGCNNNNNNGGAGGAAAACAGGCATTAGAAACAGTTCAGCGCTTGCTCCCG GTACTCTGTCAGGCACACGGTCTA
block4	CGCTTGCTCCCGGTACTCTGTCAGGCACACGGTCTAACTCCGGAACAGGTCGTA GCCATTGCTTCCNNNNNNGGCGGCAAACAGGCGCTAGAGACCGTCCAGAGGCTC TTGCCTGTGTTATGCCAGGCACATGGCCTCACCCCGGAGCAGGTCGTTGCCATC GCCAGTNNNNNNGGCGGAAAGCAAGCTCTCGAAACAGTACAACGGCTGTTGCCA GTCCTATGTCAAGCTCATGGACTG
block5	CGGCTGTTGCCAGTCCTATGTCAAGCTCATGGACTGACGCCCGAGCAGGTAGTG GCAATCGCATCTNNNNNNGGAGGTAAACAAGCACTCGAGACTGTCCAAAGATTG TTACCCGTACTATGCCAAGCGCATGGTTTAACCCCAGAGCAAGTTGTGGCTATT GCATCTNNNNNNGGTGGCAAACAAGCCTTGGAGACCGTGCAACGATTACTGCCT GTCTTATGTCAGGCCCATGGCCTT
block6	CGATTACTGCCTGTCTTATGTCAGGCCCATGGCCTTACTCCTGAGCAGGTGGTC GCTATCGCCAGCNNNNNNGGGGGCAAGCAAGCACTGGAAACAGTCCAGCGTTTG CTTCCAGTACTTTGTCAGGCGCATGGATTGACACCGGAACAAGTGGTGGCTATA GCCTCANNNNNNGGAGGAAAGCAGGCGCTGGAAACCGTCCAACGTCTTTTACCG GTGCTTTGCCAGGCGCACGGGCTC
block6'	CGATTACTGCCTGTCTTATGTCAGGCCCATGGCCTTACTCCTGAGCAAGTCGTA GCTATCGCCAGCNNNNNNGGTGGGAAACAGGCCCTGGAAACCGTACAACGTCTC CTCCCAGTACTTTGTCAAGCACACGGGTTGACACCGGAACAAGTGGTGGCGATT GCGTCCNNNNNNGGAGGCAAGCAGGCACTGGAGACCGTCCAACGGCTTCTTCCG GTTCTTTGCCAGGCTCATGGGCTC
block7	CGGCTTCTTCCGGTTCTTTGCCAGGCTCATGGGCTCACGCCAGAGCAGGTGGTA GCAATAGCGTCGNNNNNNGGTGGTAAGCAAGCGCTTGAAACGGTCCAGCGTCTT CTGCCGGTGTTGTGCCAGGCGCACGGACTCACACCAGAACAAGTGGTTGCTATT GCTAGTNNNNNNGGTGGAAAGCAGGCCCTCGAGACGGTGCAGAGGTTACTTCCC GTCCTCTGTCAAGCGCACGGCCTC

Table S2. re-TALE blocks primer sequences

block0-F	CGCAATGCGCTCACGGGAGCACCCCTCAACctAACCCCTGAACAGGT*A*G
block0-R	GAGACCATGCGCCTGACAAAGTACAGGCAGCAGTCTCTGAACAG*T*T
block1'-F	TGGCGCAATGCGCTCACGGGAGCACCCCTCA*A*C
block1-F	AGACTGCTGCCTGTACTTTGTCAGGCGCATGGTCTCACCCCCGAACA*G*G
block1-R/block1'-R	TAACCCATGTGCTTGGCACAGAACGGGCAACAACCTTTGAACCG*T*T
block2-F	AGGTTGTTGCCCGTTCTGTGCCAAGCACATGGGTTAACACCCgaac*a*a
blcok2-R	AGGCGTCAAGCCGTGGGCTTGACACAAAACAGGAAGGAGTCTCTGCACAG*T*t
block3-F	AGACTCCTTCCTGTTTTGTGTCAAGCCCACGGCTTGACGCCTG*A*G
block3-R	TAGACCGTGTGCCTGACAGAGTACCGGGAGCAAGCGCT*G*A
block4-F	CGCTTGCTCCCGGTACTCTGTCAGGCACACGGTCTAA*C*T

block4-R	CAGTCCATGAGCTTGACATAGGACTGGCAACAGCCGTT*G*T
block5-F	CGGCTGTTGCCAGTCCTATGTCAAGCTCATGGACTGA*C*G
block5-R	AAGGCCATGGGCCTGACATAAGACAGGCAGTAATCGTT*G*C
block6-F	CGATTACTGCCTGTCTTATGTCAGGCCCATGGCCTTA*C*T
block6-R	GAGCCCGTGCGCCTGGCAAAGCACCGGTAAAAGACGTTGGA*C*G
block6'-F	CGATTACTGCCTGTCTTATGTCAGGCCCATGGCCTTACTCCTGAGCAA*G*T
block6'-R	GAGCCCATGAGCCTGGCAAAGAACCGGAAGAAGCCGTT*G*G
block7-F	CGGCTTCTTCCGGTTCTTTGCCAGGCTCATGGGCTCACGCCAGAGCAGG*T*G
blcok7-R	GAGGCCGTGCGCTTGACAGAGGACGGGAAGTAACCTCT*G*C

Table S3. re-TALEN pairs and Cas9-gRNAs targeting *CCR5*

# targeting site	re-TALENs pair targeting site (start) /chr3:	re-TALENs pair targeting site (end) /chr3:	re-TALEN-L targeting sequence	re-TALEN-R targeting sequence	gRNA targeting sequence	gRNA targeting sequence start position
1	46409942	46409993	TCCCCACTTTCTTGTGAA	TAACCACTCAGGACAGGG	CACTTTCTTGTGAATCCTT	46409946
2	46410227	46410278	TCACACAGCAAGTCAGCA	TAGCGGAGCAGGCTCGGA	TGGGCTAGCGGAGCAGGCT	46410264
3	46411260	46411311	TACCCAGACGAGAAAGCT	TCAGACTGCCAAGCTTGA	ACCCAGACGAGAAAGCTGA	46411261
4	46411464	46411515	TCTTGTGGCTCGGGAGTA	TATTGTCAGCAGAGCTGA	AGAGGGCATCTTGTGGCTC	46411456
5	46411517	46411568	TTGAGATTTTCAGATGTC	TATACAGTCATATCAAGC	ATCAAGCTCTCTTGGCGGT	46411538
6	46411634	46411685	TTCAGATAGATTATATCT	TGCCAGATACATAGGTGG	GCTTCAGATAGATTATATC	46411632

7	46412396	46412447	TTATACTGTCTATATGAT	TCAGCTCTTCTGGCCAGA	ACGGATGTCTCAGCTCTTC	46412437
8	46412432	46412483	TGGCCAGAAGAGCTGAGA	TTACCGGGGAGAGTTTCT	CCGGGGAGAGTTTCTTGTA	46412461
9	46412750	46412801	TTTGCAGAGAGATGAGTC	TTAGCAGAAGATAAGATT	GAAATCTTATCTTCTGCTA	46412782
10	46413152	46413203	TATAAGACTAAACTACCC	TCGTCTGCCACCACAGAT	AATGCATGACATTCATCTG	46413172
11	46414305	46414356	TAAAACAGTTTGCATTCA	TATAAAGTCCTAGAATGT	AACAGTTTGCATTCATGGA	46414308
12	46414608	46414659	TGGCCATCTCTGACCTGT	TAGTGAGCCCAGAAGGGG	CCAGAAGGGGACAGTAAGA	46414632
13	46414768	46414820	TAGGTACCTGGCTGTCGT	TGACCGTCCTGGCTTTTA	CTGACAATCGATAGGTACC	46414757
14	46415017	46415068	TGTCATGGTCATCTGCTA	TCGACACCGAAGCAGAGT	ACACCGAAGCAGAGTTTTT	46415046
15	46420034	46420084	TGCCCCCGCGAGGCCACA	TCTGGAAGTTGAACACCC	GGAAGTTGAACACCCTTGC	46420062

Table S4. HDR and NHEJ efficiency of re-TALENs and Cas9-gRNAs targeting *CCR5*

# targeting site	cell type	HDR (reTALEN) (%)	NHEJ (reTALE) (%)	HDR detection limit based on Information analysis	NHEJ (Cas9-gRNA)	HDR (Cas9-gRNA)
1	PGP1-iPS	0.06%	0.80%	0.04%	0.58%	0.38%
2	PGP1-iPS	0.48%	0.26%	0.01%	16.02%	3.71%
3	PGP1-iPS	1.71%	0.07%	0.03%	3.44%	3.20%
4	PGP1-iPS	0.02%	1.20%	0.02%*	1.50%	0.14%

5	PGP1-iPS	0.80%	0.04%	0.00%	3.70%	0.39%
6	PGP1-iPS	0.20%	0.73%	0.00%	1.12%	0.49%
7	PGP1-iPS	0.01%	0.15%	0.01%*	1.98%	1.78%
8	PGP1-iPS	0.03%	0.00%	0.00%	1.85%	0.03%
9	PGP1-iPS	1.60%	0.06%	0.00%	0.50%	0.13%
10	PGP1-iPS	0.68%	1.25%	0.01%	8.77%	1.32%
11	PGP1-iPS	0.06%	0.27%	0.00%	0.62%	0.44%
12	PGP1-iPS	1.60%	0.03%	0.04%	0.18%	0.99%
13	PGP1-iPS	0.00%	1.47%	0.00%	0.65%	0.02%
14	PGP1-iPS	0.47%	0.13%	0.02%	2.50%	0.31%
15	PGP1-iPS	0.8	0.14	0.08%	1.50	1.10%

* The group where HDR detection limit exceeds the real HDR detected

Table S5. CCR5 targeting site PCR primer sequences

# targeting in CCR5	name	primer sequence
1	site1-F1	ACACTCTTTCCCTACACGACGCTCTTCCGATCTCGTGATTTTGCAGTGTGCGTTACTCC
	site1-F2	ACACTCTTTCCCTACACGACGCTCTTCCGATCTACATCGTTTGCAGTGTGCGTTACTCC
	site1-F3	ACACTCTTTCCCTACACGACGCTCTTCCGATCTGCCTAATTTGCAGTGTGCGTTACTCC
	site1-F4	ACACTCTTTCCCTACACGACGCTCTTCCGATCTTGGTCATTTGCAGTGTGCGTTACTCC
	site1-R	CTCGGCATTCCTGCTGAACCGCTCTTCCGATCTCCAAGCAACTAAGTCACAGCA
2	Site2-F1	ACACTCTTTCCCTACACGACGCTCTTCCGATCTCGTGATATGAGGAAATGGAAGCTTG
	Site2-F2	ACACTCTTTCCCTACACGACGCTCTTCCGATCTACATCGATGAGGAAATGGAAGCTTG
	Site2-F3	ACACTCTTTCCCTACACGACGCTCTTCCGATCTGCCTAAATGAGGAAATGGAAGCTTG
	Site2-F4	ACACTCTTTCCCTACACGACGCTCTTCCGATCTTGGTCAATGAGGAAATGGAAGCTTG
	Site2-R	CTCGGCATTCCTGCTGAACCGCTCTTCCGATCTCATTAGGGTATTGGAGGA
3	site3-F1	ACACTCTTTCCCTACACGACGCTCTTCCGATCTCGTGATAATCCTCCCAACAACTCAT
	site3-F2	ACACTCTTTCCCTACACGACGCTCTTCCGATCTACATCGAATCCTCCCAACAACTCAT
	site3-F3	ACACTCTTTCCCTACACGACGCTCTTCCGATCTGCCTAAAATCCTCCCAACAACTCAT
	site3-F4	ACACTCTTTCCCTACACGACGCTCTTCCGATCTTGGTCAAATCCTCCCAACAACTCAT
	site3_R	CTCGGCATTCCTGCTGAACCGCTCTTCCGATCTCCCAATCCTACAGAGGCAG
4	site4-F1	ACACTCTTTCCCTACACGACGCTCTTCCGATCTCGTGATAAGCCAAAGCTTTTTATTC
	site4-F2	ACACTCTTTCCCTACACGACGCTCTTCCGATCTACATCGAAGCCAAAGCTTTTTATTC

	site4-F3	ACACTCTTTCCCTACACGACGCTCTTCCGATCTGCCTAAAAGCCAAAGCTTTTTATTC
	site4-F4	ACACTCTTTCCCTACACGACGCTCTTCCGATCTTGGTCAAAGCCAAAGCTTTTTATTC
	site4_R	ACACTCTTTCCCTACACGACGCTCTTCCGATCTAAGCCAAAGCTTTTTATTCT
5	site5-F1	ACACTCTTTCCCTACACGACGCTCTTCCGATCTCGTGATATCTTGTGGCTCGGGAGTAG
	site5-F2	ACACTCTTTCCCTACACGACGCTCTTCCGATCTACATCGATCTTGTGGCTCGGGAGTAG
	site5-R	CTCGGCATTCCTGCTGAACCGCTCTTCCGATCTTGGCAGGATTCTTCACTCCA
6	site6-F1	ACACTCTTTCCCTACACGACGCTCTTCCGATCTCGTGATCTATTTTGTTGCCCTTCAAA
	site6-F2	ACACTCTTTCCCTACACGACGCTCTTCCGATCTACATCGCTATTTTGTTGCCCTTCAAA
	site6-R	CTCGGCATTCCTGCTGAACCGCTCTTCCGATCTAACCTGAACTTGACCATATACT
7	site7-F1	ACACTCTTTCCCTACACGACGCTCTTCCGATCTCGTGATCAGCTGAGAGGTTACTTACC
	site7-F2	ACACTCTTTCCCTACACGACGCTCTTCCGATCTACATCGCAGCTGAGAGGTTACTTACC
	site7-R	CTCGGCATTCCTGCTGAACCGCTCTTCCGATCTAATGATTTAACTCCACCCTC
8	site8-F1	ACACTCTTTCCCTACACGACGCTCTTCCGATCTCGTGATACTCCACCCTCCTTCAAAAGA
	site8-F2	ACACTCTTTCCCTACACGACGCTCTTCCGATCTACATCGACTCCACCCTCCTTCAAAAGA
	site8-R	CTCGGCATTCCTGCTGAACCGCTCTTCCGATCTTGGTGTTTGCCAAATGTCT
9	site9_F1	ACACTCTTTCCCTACACGACGCTCTTCCGATCTCGTGATGGGCACATATTCAGAAGGCA
	site9_F2	ACACTCTTTCCCTACACGACGCTCTTCCGATCTACATCGGGGCACATATTCAGAAGGCA
	site9_R	CTCGGCATTCCTGCTGAACCGCTCTTCCGATCTAGTGAAAGACTTTAAAGGGAGCA
10	site10-F1	ACACTCTTTCCCTACACGACGCTCTTCCGATCTCGTGATCACAATTAAGAGTTGTCATA
	site10-F2	ACACTCTTTCCCTACACGACGCTCTTCCGATCTACATCGCACAATTAAGAGTTGTCATA
	site10-R	CTCGGCATTCCTGCTGAACCGCTCTTCCGATCTCTCAGCTAGAGCAGCTGAAC
11	site11-F1	CTCGGCATTCCTGCTGAACCGCTCTTCCGATCTGACACTTGATAATCCATC
	site11-F2	ACACTCTTTCCCTACACGACGCTCTTCCGATCTACATCGTCAATGTAGACATCTATGTAG
	site11-R	ACACTCTTTCCCTACACGACGCTCTTCCGATCTCGTGATTCAATGTAGACATCTATGTAG
12	site12-F1	ACACTCTTTCCCTACACGACGCTCTTCCGATCTCGTGATACTGCAAAAGGCTGAAGAGC
	site12-F2	ACACTCTTTCCCTACACGACGCTCTTCCGATCTACATCGACTGCAAAAGGCTGAAGAGC
	site12-F3	ACACTCTTTCCCTACACGACGCTCTTCCGATCTGCCTAAACTGCAAAAGGCTGAAGAGC
	site12-F4	ACACTCTTTCCCTACACGACGCTCTTCCGATCTTGGTCAACTGCAAAAGGCTGAAGAGC
	site12-R	CTCGGCATTCCTGCTGAACCGCTCTTCCGATCTGCCTATAAAATAGAGCCCTGTCAA
13	site13-F1	ACACTCTTTCCCTACACGACGCTCTTCCGATCTCGTGATCTCTATTTTATAGGCTTCTTC
	site13-F2	ACACTCTTTCCCTACACGACGCTCTTCCGATCTACATCGCTCTATTTTATAGGCTTCTTC
	site13-R	CTCGGCATTCCTGCTGAACCGCTCTTCCGATCTAGCCACCACCCAAGTGATC
14	site14-F1	ACACTCTTTCCCTACACGACGCTCTTCCGATCTACATCGTTCCAGACATTAAAGATAGTC
	site14-F2	ACACTCTTTCCCTACACGACGCTCTTCCGATCTCGTGATTTCCAGACATTAAAGATAGTC
	site14-R	CTCGGCATTCCTGCTGAACCGCTCTTCCGATCTAATCATGATGGTGAAGATAAG
15	site15-F1	ACACTCTTTCCCTACACGACGCTCTTCCGATCTCGTGATCCGGCAGAGACAAACATTAAA
	site15-F2	ACACTCTTTCCCTACACGACGCTCTTCCGATCTCCGGCAGAGACAAACATTAAA
	site15-R	CTCGGCATTCCTGCTGAACCGCTCTTCCGATCTAGCTAGGAAGCCATGGCAAG
illumina adaptor	PE-PCR-F	AATGATACGGCGACCACCGAGATCTACACTCTTTCCCTACACgac*g*c
	PE-PCR-R	CAAGCAGAAGACGGCATACGAGATCGGTCTCGGCATTCCTGCTGAACc*g*c

Multiplex sequencing PCR primer		
3	site3-M-F	ACACTCTTTCCCTACACGACGCTCTTCCGATCTAGTGCATAGTATGTGCTAGATGCTG
	site3-M-R	GTGACTGGAGTTCAGACGTGTGCTCTTCCGATCTTGATCTCTAAGAAGGCAAATGAGAC
illumina adaptor	Index-PCR	CAAGCAGAAGACGGCATACGAGAT$N_1N_2N_3N_4N_5N_6$GTGACTGGAGTTCAGACGTGTGCTCTTCCGATCT
	universal-PCR	AATGATACGGCGACCACCGAGATCTACACTCTTTCCCTACACGACGCTCTTCCGATCT

*index-PCR primers are purchased from epicentre (ScriptSeq™ Index PCR Primers)

Table S6. ssODN design for studying ssODN-mediated genome editing

Figure 3b	Distance between the secondary mutation and DSB	90-*1	CTACTGTCATTCAGGGCAATACCCAGACGAGAAAGCTGAGGGTATAACAGGTTTCAAGCTTGGCAGTCTGACTACAGAGGCCACTGGCTT
		90-*2	CTACTGTCATTCAGCCCAATACCCTAACGAGAAAGCTGAGGGTATAACAGGTTTCAAGCTTGGCAGTCTGACTACAGAGGCCACTGGCTT
		90-*3	CTACTGTCATTCAGCCCAATACCCAGACGAGAAAAGTGAGGGTATAACAGGTTTCAAGCTTGGCAGTCTGACTACAGAGGCCACTGGCTT
		90M-0	CTACTGTCATTCAGCCCAATACCCAGACGAGAAAGCTGAGGGTATAACAGGTTTCAAGCTTGGCAGTCTGACTACAGAGGCCACTGGCTT
		90-*4	CTACTGTCATTCAGCCCAATACCCAGACGAGAAAGCTGAGGGTATAACAGGTTTGTAGCTTGGCAGTCTGACTACAGAGGCCACTGGCTT
		90-*5	CTACTGTCATTCAGCCCAATACCCAGACGAGAAAGCTGAGGGTATAACAGGTTTCAAGCTTGGCTCTCTGACTACAGAGGCCACTGGCTT
		90-*6	CTACTGTCATTCAGCCCAATACCCAGACGAGAAAGCTGAGGGTATAACAGGTTTCAAGCTTGGCAGTCTGACTAGTGAGGCCACTGGCTT
Figure 3c	distance between ssODN and the DSB	L670bp_90M	CACTTTATATTTCCCTGCTTAAACAGTCCCCCGAGGGTGGGTGCGGAAAAGGCTCTACACTTGTTATCATTCCCTCTCCACCACAGGCAT
		L570bp_90M	TTTGTATTTGGGTTTTTTTAAAACCTCCACTCTACAGTTAAGAATTCTAAGGCACAGAGCTTCAATAATTTGGTCAGAGCCAAGTAGCAG
		L480bp_90M	GGAGGTTAAACCCAGCAGCATGACTGCAGTTCTTAATCAATGCCCCTTGAATTGCACATATGGGATGAACTAGAACATTTTCTCGATGAT

		L394bp_90M	CTCGATGATTCGCTGTCCTTGTTATGATTATGTTACTGAGCTCTACTGTAGCACAGACATATGTCCCTATATGGGGCGGGGGTGGGGGTG
		L290bp_90M	GGTGTCTTGATCGCTGGGCTATTTCTATACTGTTCTGGCTTTTCGGAAGCAGTCATTTCTTTCTATTCTCCAAGCACCAGCAATTAGCTT
		L200bp_90M	GCTTCTAGTTTGCTGAAACTAATCTGCTATAGACAGAGACTCCGACGAACCAATTTTATTAGGATTTGATCAAATAAACTCTCTCTGACA
		L114bp_90M	GAAAGAGTAACTAAGAGTTTGATGTTTACTGAGTGCATAGTATGCACTAGATGCTGGCCGTGGATGCCTCATAGAATCCTCCCAACAACT
		L45bp_90M	GCTAGATGCTGGCCGTGGATGCCTCATAGAATCCTCCCAACAACCGATGAAATGACTACTGTCATTCAGCCCAATACCCAGACGAGAAAG
		R40bp_90M	ACAGGTTTCAAGCTTGGCAGTCTGACTACAGAGGCCACTGGCTTTACCCCTGGGTTAGTCTGCCTCTGTAGGATTGGGGGCACGTAATTT
		R100bp_90M	TTAGTCTGCCTCTGTAGGATTGGGGGCACGTAATTTTGCTGTTTAAGGTCTCATTTGCCTTCTTAGAGATCACAAGCCAAAGCTTTTTAT
		R200bp_90M	GGAAGCCCAGAGGGCATCTTGTGGCTCGGGAGTAGCTCTCTGCTACCTTCTCAGCTCTGCTGACAATACTTGAGATTTTCAGATGTCACC
		R261bp_90M	TCAGCTCTGCTGACAATACTTGAGATTTTCAGATGTCACCAACCAGCAAGAGAGCTTGATATGACTGTATATAGTATAGTCATAAAGAAC
		R322bp_90M	CATAAAGAACCTGAACTTGACCATATACTTATGTCATGTGGAAATCTTCTCATAGCTTCAGATAGATTATATCTGGAGTGAAGAATCCTG
		R375M_90M	GTGGAAAATTTCTCATAGCTTCAGATAGATTATATCTGGAGTGAGCAATCCTGCCACCTATGTATCTGGCATAGTGTGAGTCCTCATAAA
		R448bp_90M	GGTTTGAAGGGCAACAAAATAGTGAACAGAGTGAAAATCCCCACCTAGATCCTGGGTCCAGAAAAAGATGGGAAACCTGTTTAGCTCACC
		Complement-30mer	GGCCACTAGGGACAAAATTGGTGAcagaaa
Figure 3d	ssODN length and orientation for Cas9-gRNA targetin	Complement-50mer	CCCACAGTGGGGCCACTAGGGACAAAATTGGTGAcagaaaagccccatcc
		Complement-70mer	TCCCCTCCACCCCACAGTGGGGCCACTAGGGACAAAATTGGTGAcagaaaagccccatccttaggcctcc
		Complement-90mer	cttTTATCTGTCCCCTCCACCCCACAGTGGGGCCACTAGGGACAAAATTGGTGAcagaaaagccccatccttaggcctcctccttcctag

	g	Complement-110mer	gttctgggtactttTTATCTGTCCCCTCCACCCCACAGTGGGGCCACTAGGGACAAAATTGGTGAcagaaaagccccatccttaggcctcctccttcctagtctcctgata
		Non-complement-30mer	TTTCTGTCACCAATGGTGTCCCTAGTGGCC
		Non-complement-50mer	GGATGGGGCTTTTCTGTCACCAATGGTGTCCCTAGTGGCCCCACTGTGGG
		Non-complement-70mer	GGAGGCCTAAGGATGGGGCTTTTCTGTCACCAATGGTGTCCCTAGTGGCCCCACTGTGGGGTGGAGGGGA
		Non-complement-90mer	CTAGGAAGGAGGAGGCCTAAGGATGGGGCTTTTCTGTCACCAATGGTGTCCCTAGTGGCCCCACTGTGGGGTGGAGGGGACAGATAAAAG
		Non-complement-110mer	TATCAGGAGACTAGGAAGGAGGAGGCCTAAGGATGGGGCTTTTCTGTCACCAATGGTGTCCCTAGTGGCCCCACTGTGGGGTGGAGGGGACAGATAAAAGTACCCAGAAC
Figure 2c	ssODN donor for Cas9-gRNA targeting CCR5	Cas9-gRNA-CCR5-1	TTCTAGTAACCACTCAGGACAGGGGGGTTCAGCCCAAAAATTCACAAGAAAGTGGGGACCCATGGGAAAT
		Cas9-gRNA-CCR5-2	CAGCAAGTCAGCAGCACAGCGTGTGTGACTCCGAGGGTGCTCCGCTAGCCCACATTGCCCTCTGGGGGTG
		Cas9-gRNA-CCR5-3	GTCAGACTGCCAAGCTTGAAACCTGTCTTACCCTCTACTTTCTCGTCTGGGTATTGGGCTGAATGACAGT
		Cas9-gRNA-CCR5-4	CAGAGCTGAGAAGACAGCAGAGAGCTACTCCCGAAGCACAAGATGCCCTCTGGGCTTCCGTGACCTTGGC
		Cas9-gRNA-CCR5-5	CTGACAATACTTGAGATTTTCAGATGTCACCAACGACCAAGAGAGCTTGATATGACTGTATATAGTATAG
		Cas9-gRNA-CCR5-6	CAGATACATAGGTGGCAGGATTCTTCACTCCAGACTTAATCTATCTGAAGCTATGAGAAATTTTCCACAT
		Cas9-gRNA-CCR5-7	TATATGATTGATTTGCACAGCTCATCTGGCCAGATAAGCTGAGACATCCGTTCCCCTACAAGAAACTCTC
		Cas9-gRNA-CCR5-8	ATCTGGCCAGAAGAGCTGAGACATCCGTTCCCCTTGAAGAAACTCTCCCCGGTAAGTAACCTCTCAGCTG
		Cas9-gRNA-CCR5-9	AGGCATCTCACTGGAGAGGGTTTAGTTCTCCTTAAGAGAAGATAAGATTTCAAGAGGGAAGCTAAGACTC
		Cas9-gRNA-CCR5-10	ATAATATAATAAAAAATGTTTCGTCTGCCACCACTAATGAATGTCATGCATTCTGGGTAGTTTAGTCTTA
		Cas9-gRNA-CCR5-11	TTTATAAAGTCCTAGAATGTATTTAGTTGCCCTCGTTGAATGCAAACTGTTTTATACATCAATAGGTTTT

	Cas9-gRNA-CCR5-12	GCTCAACCTGGCCATCTCTGACCTGTTTTTCCTTCCCACTGTCCCCTTCTGGGCTCACTATGCTGCCGCC
	Cas9-gRNA-CCR5-13	TTTTAAAGCAAACACAGCATGGACGACAGCCAGGCTCCTATCGATTGTCAGGAGGATGATGAAGAAGATT
	Cas9-gRNA-CCR5-14	GCTTGTCATGGTCATCTGCTACTCGGGAATCCTAATTACTCTGCTTCGGTGTCGAAATGAGAAGAAGAGG
	Cas9-gRNA-CCR5-15	ATACTGCCCCCGCGAGGCCACATTGGCAAACCAGCTTGGGTGTTCAACTTCCAGACTTGGCCATGGAGAA
ssODN donor for reTALENs targeting CCR5	reTALEN-CCR5-1	CTGAAGAATTTCCCATGGGTCCCCACTTTCTTGTGAATCCTTGGAGTGAACCCCCCTGTCCTGAGTGGTTACTAGAACACACCTCTGGAC
	reTALEN-CCR5-2	TGGAAGTATCTTGCCGAGGTCACACAGCAAGTCAGCAGCACAGCCAGTGTGACTCCGAGCCTGCTCCGCTAGCCCACATTGCCCTCTGGG
	reTALEN-CCR5-3	CTACTGTCATTCAGCCCAATACCCAGACGAGAAAGCTGAGGGTATAACAGGTTTCAAGCTTGGCAGTCTGACTACAGAGGCCACTGGCTT
	reTALEN-CCR5-4	GGAAGCCCAGAGGGCATCTTGTGGCTCGGGAGTAGCTCTCTGCTACCTTCTCAGCTCTGCTGACAATACTTGAGATTTTCAGATGTCACC
	reTALEN-CCR5-5	TCAGCTCTGCTGACAATACTTGAGATTTTCAGATGTCACCAACGCCCAAGAGAGCTTGATATGACTGTATATAGTATAGTCATAAAGAAC
	reTALEN-CCR5-6	GTGGAAAATTTCTCATAGCTTCAGATAGATTATATCTGGAGTGAGCAATCCTGCCACCTATGTATCTGGCATAGTGTGAGTCCTCATAAA
	reTALEN-CCR5-7	GAAACAGCATTTCCTACTTTTATACTGTCTATATGATTGATTTGGTCAGCTCATCTGGCCAGAAGAGCTGAGACATCCGTTCCCCTACAA
	reTALEN-CCR5-8	TTGATTTGCACAGCTCATCTGGCCAGAAGAGCTGAGACATCCGTATCCCTACAAGAAACTCTCCCCGGTAAGTAACCTCTCAGCTGCTTG
	reTALEN-CCR5-9	GGAGAGGGTTTAGTTCTCCTTAGCAGAAGATAAGATTTCAAGATGAGAGCTAAGACTCATCTCTCTGCAAATCTTTCTTTTGAGAGGTAA
	reTALEN-CCR5-10	TAATATAATAAAAAATGTTTCGTCTGCCACCACAGATGAATGTCGAGCATTCTGGGTAGTTTAGTCTTATAACCAGCTGTCTTGCCTAGT
	reTALEN-CCR5-11	TTAAAAACCTATTGATGTATAAAACAGTTTGCATTCATGGAGGGTGACTAAATACATTCTAGGACTTTATAAAAGATCACTTTTTATTTA

	reTALEN-CCR5-12	GACATCTACCTGCTCAACCTGGCCATCTCTGACCTGTTTTTCCTATTTACTGTCCCCTTCTGGGCTCACTATGCTGCCGCCCAGTGGGAC
	reTALEN-CCR5-13	TCATCCTCCTGACAATCGATAGGTACCTGGCTGTCGTCCATGCTACGTTTGCTTTAAAAGCCAGGACGGTCACCTTTGGGGTGGTGACAA
	reTALEN-CCR5-14	GGCTGGTCCTGCCGCTGCTTGTCATGGTCATCTGCTACTCGGGAGACCTAAAAACTCTGCTTCGGTGTCGAAATGAGAAGAAGAGGCACA
	reTALEN-CCR5-15	GGCAAGCCTTGGGTCATACTGCCCCCGCGAGGCCACATTGGCAAGTCAGCAAGGGTGTTCAACTTCCAGACTTGGCCATGGAGAAGACAT

Supplementary Sequence 1

re-TALE (16.5) sequence

CTAACCCCTGAACAGGTAGTCGCTATAGCTTCAAATATCGGGGGCAAGCAAGCACTTGAGACCGTTCAAC
GACTCCTGCCAGTGCTCTGCCAAGCCCATGGATTGACTCCGGAGCAAGTCGTCGCGATCGCGAGCAACG
GCGGGGGGAAGCAGGCGCTGGAAACTGTTCAGAGACTGCTGCCTGTACTTTGTCAGGCGCATGGTCTCA
CCCCCGAACAGGTTGTCGCAATAGCAAGTAATATAGGCGGTAAGCAAGCCCTAGAGACTGTGCAACGCC
TGCTCCCCGTGCTGTGTCAGGCTCACGGTCTGACACCTGAACAAGTTGTCGCGATAGCCAGTCACGACGG
GGGAAAACAAGCTCTAGAAACGGTTCAAAGGTTGTTGCCCGTTCTGTGCCAAGCACATGGGTTAACACC
CGAACAAGTAGTAGCGATAGCGTCAAATAACGGGGGTAAACAGGCTTTGGAGACGGTACAGCGGTTAT
TGCCGGTCCTCTGCCAGGCCCACGGACTTACGCCAGAACAGGTGGTTGCAATTGCCTCCAACATCGGCGG
GAAACAAGCGTTGGAAACTGTGCAGAGACTCCTTCCTGTTTTGTGTCAAGCCCACGGCTTGACGCCTGAG
CAGGTTGTGGCCATCGCTAGCCACGACGGAGGGAAGCAGGCTCTTGAAACCGTACAGCGACTTCTCCCA
GTTTTGTGCCAAGCTCACGGGCTAACCCCCGAGCAAGTAGTTGCCATAGCAAGCAACGGAGGAGGAAAA
CAGGCATTAGAAACAGTTCAGCGCTTGCTCCCGGTACTCTGTCAGGCACACGGTCTAACTCCGGAACAGG
TCGTAGCCATTGCTTCCCATGATGGCGGCAAACAGGCGCTAGAGACAGTCCAGAGGCTCTTGCCTGTGTT
ATGCCAGGCACATGGCCTCACCCCGGAGCAGGTCGTTGCCATCGCCAGTAATATCGGCGGAAAGCAAGC
TCTCGAAACAGTACAACGGCTGTTGCCAGTCCTATGTCAAGCTCATGGACTGACGCCCGAGCAGGTAGTG
GCAATCGCATCTCACGATGGAGGTAAACAAGCACTCGAGACTGTCCAAAGATTGTTACCCGTACTATGCC
AAGCGCATGGTTTAACCCCAGAGCAAGTTGTGGCTATTGCATCTAACGGCGGTGGCAAACAAGCCTTGG
AGACAGTGCAACGATTACTGCCTGTCTTATGTCAGGCCCATGGCCTTACTCCTGAGCAAGTCGTAGCTAT
CGCCAGCAACATAGGTGGGAAACAGGCCCTGGAAACCGTACAACGTCTCCTCCCAGTACTTTGTCAAGCA
CACGGGTTGACACCGGAACAAGTGGTGGCGATTGCGTCCAACGGCGGAGGCAAGCAGGCACTGGAGAC
CGTCCAACGGCTTCTTCCGGTTCTTTGCCAGGCTCATGGGCTCACGCCAGAGCAGGTGGTAGCAATAGCG
TCGAACATCGGTGGTAAGCAAGCGCTTGAAACGGTCCAGCGTCTTCTGCCGGTGTTGTGCCAGGCGCAC
GGACTCACACCAGAACAAGTGGTTGCTATTGCTAGTAACAACGGTGGAAAGCAGGCCCTCGAGACGGTG
CAGAGGTTACTTCCCGTCCTCTGTCAAGCGCACGGCCTCACTCCAGAGCAAGTGGTTGCGATCGCTTCAA
ACAATGGTGGAAGACCTGCCCTGGAA

Supplementary Sequence 2

re-TALEN-backbone sequence

(purple: re-TALE-N; red: SapI site; green: 0.5 monomer; blue: re-TALEN-C; orange: Fok I)

ATGTCGCGGACCCGGCTCCCTTCCCCACCCGCACCCAGCCCAGCGTTTTCGGCCGACTCGTTCTCAGACCT
GCTTAGGCAGTTCGACCCCTCACTGTTTAACACATCGTTGTTCGACTCCCTTCCTCCGTTTGGGGCGCACC
ATACGGAGGCGGCCACCGGGGAGTGGGATGAGGTGCAGTCGGGATTGAGAGCTGCGGATGCACCACC
CCCAACCATGCGGGTGGCCGTCACCGCTGCCCGACCGCCGAGGGCGAAGCCCGCACCAAGGCGGAGGG
CAGCGCAACCGTCCGACGCAAGCCCCGCAGCGCAAGTAGATTTGAGAACTTTGGGATATTCACAGCAGC
AGCAGGAAAAGATCAAGCCCAAAGTGAGGTCGACAGTCGCGCAGCATCACGAAGCGCTGGTGGGTCAT
GGGTTTACACATGCCCACATCGTAGCCTTGTCGCAGCACCCTGCAGCCCTTGGCACGGTCGCCGTCAAGT
ACCAGGACATGATTGCGGCGTTGCCGGAAGCCACACATGAGGCGATCGTCGGTGTGGGGAAACAGTGG
AGCGGAGCCCGAGCGCTTGAGGCCCTGTTGACGGTCGCGGGAGAGCTGAGAGGGCCTCCCCTTCAGCT
GGACACGGGCCAGTTGCTGAAGATCGCGAAGCGGGGAGGAGTCACGGCGGTCGAGGCGGTGCACGCG
TGGCGCAATGCGCTCACGGGAGCACCCCTCAACAGTTCACGCTGACAGAGACCGCGGCCGCATTAGGCA
CCCCAGGCTTTACACTTTATGCTTCCGGCTCGTATAATGTGTGGATTTTGAGTTAGGATCCGTCGAGATTT
TCAGGAGCTAAGGAAGCTAAAATGGAGAAAAAAATCACTGGATATACCACCGTTGATATATCCCAATGG
CATCGTAAAGAACATTTTGAGGCATTTCAGTCAGTTGCTCAATGTACCTATAACCAGACCGTTCAGCTGGA
TATTACGGCCTTTTTAAAGACCGTAAAGAAAAATAAGCACAAGTTTTATCCGGCCTTTATTCACATTCTTG
CCCGCCTGATGAATGCTCATCCGGAATTCCGTATGGCAATGAAAGACGGTGAGCTGGTGATATGGGATA
GTGTTCACCCTTGTTACACCGTTTTCCATGAGCAAACTGAAACGTTTTCATCGCTCTGGAGTGAATACCAC
GACGATTTCCGGCAGTTTCTACACATATATTCGCAAGATGTGGCGTGTTACGGTGAAAACCTGGCCTATT
TCCCTAAAGGGTTTATTGAGAATATGTTTTTCGTCTCAGCCAATCCCTGGGTGAGTTTCACCAGTTTTGATT
TAAACGTGGCCAATATGGACAACTTCTTCGCCCCCGTTTTCACCATGGGCAAATATTATACGCAAGGCGA
CAAGGTGCTGATGCCGCTGGCGATTCAGGTTCATCATGCCGTTTGTGATGGCTTCCATGTCGGCAGAATG
CTTAATGAATTACAACAGTACTGCGATGAGTGGCAGGGCGGGGCGTAAAGATCTGGATCCGGCTTACTA
AAAGCCAGATAACAGTATGCGTATTTGCGCGCTGATTTTTGCGGTATAAGAATATATACTGATATGTATA
CCCGAAGTATGTCAAAAAGAGGTATGCTATGAAGCAGCGTATTACAGTGACAGTTGACAGCGACAGCTA
TCAGTTGCTCAAGGCATATATGATGTCAATATCTCCGGTCTGGTAAGCACAACCATGCAGAATGAAGCCC
GTCGTCTGCGTGCCGAACGCTGGAAAGCGGAAAATCAGGAAGGGATGGCTGAGGTCGCCCGGTTTATT
GAAATGAACGGCTCTTTTGCTGACGAGAACAGGGGCTGGTGAAATGCAGTTTAAGGTTTACACCTATAA
AAGAGAGAGCCGTTATCGTCTGTTTGTGGATGTACAGAGTGATATTATTGACACGCCCGGGCGACGGAT
GGTGATCCCCCTGGCCAGTGCACGTCTGCTGTCAGATAAAGTCTCCCGTGAACTTTACCCGGTGGTGCAT
ATCGGGGATGAAAGCTGGCGCATGATGACCACCGATATGGCCAGTGTGCCGGTCTCCGTTATCGGGGAA
GAAGTGGCTGATCTCAGCCACCGCGAAAATGACATCAAAAACGCCATTAACCTGATGTTCTGGGGAATAT
AAATGTCAGGCTCCCTTATACACAGCCAGTCTGCAGGTCGACGGTCTCGCTCTTCGAAGGTTACTTCCCGT
CCTCTGTCAAGCGCACGGCCTCACTCCAGAGCAAGTGGTTGCGATCGCTTCAAACAACGGTGGAAGACCT
GCCCTGGAATCAATCGTGGCCCAGCTTTCGAGGCCGGACCCCGCGCTGGCCGCACTCACTAATGATCATC
TTGTAGCGCTGGCCTGCCTCGGCGGACGACCCGCCTTGGATGCGGTGAAGAAGGGGCTCCCGCACGCGC
CTGCATTGATTAAGCGGACCAACAGAAGGATTCCCGAGAGGACATCACATCGAGTGGCAGGTTCCCAAC

TCGTGAAGAGTGAACTTGAGGAGAAAAAGTCGGAGCTGCGGCACAAATTGAAATACGTACCGCATGAA
TACATCGAACTTATCGAAATTGCTAGGAACTCGACTCAAGACAGAATCCTTGAGATGAAGGTAATGGAGT
TCTTTATGAAGGTTTATGGATACCGAGGGAAGCATCTCGGTGGATCACGAAAACCCGACGGAGCAATCT
ATACGGTGGGGAGCCCGATTGATTACGGAGTGATCGTCGACACGAAAGCCTACAGCGGTGGGTACAATC
TTCCCATCGGGCAGGCAGATGAGATGCAACGTTATGTCGAAGAAAATCAGACCAGGAACAAACACATCA
ATCCAAATGAGTGGTGGAAAGTGTATCCTTCATCAGTGACCGAGTTTAAGTTTTTGTTTGTCTCTGGGCAT
TTCAAAGGCAACTATAAGGCCCAGCTCACACGGTTGAATCACATTACGAACTGCAATGGTGCGGTTTTGT
CCGTAGAGGAACTGCTCATTGGTGGAGAAATGATCAAAGCGGGAACTCTGACACTGGAAGAAGTCAGA
CGCAAGTTTAACAATGGCGAGATCAATTTCCGC

re-TALE-TF backbone sequence

(purple: re-TALE-N; red: SapI site; green: 0.5 monomer; blue: re-TALEN-C; orange: NLS-VP64; 2A-GFP is highlighted in green)

ATGTCGCGGACCCGGCTCCCTTCCCCACCCGCACCCAGCCCAGCGTTTTCGGCCGACTCGTTCTCAGACCT
GCTTAGGCAGTTCGACCCCTCACTGTTTAACACATCGTTGTTCGACTCCCTTCCTCCGTTTGGGGCGCACC
ATACGGAGGCGGCCACCGGGGAGTGGGATGAGGTGCAGTCGGGATTGAGAGCTGCGGATGCACCACC
CCCAACCATGCGGGTGGCCGTCACCGCTGCCCGACCGCCGAGGGCGAAGCCCGCACCAAGGCGGAGGG
CAGCGCAACCGTCCGACGCAAGCCCCGCAGCGCAAGTAGATTTGAGAACTTTGGGATATTCACAGCAGC
AGCAGGAAAAGATCAAGCCCAAAGTGAGGTCGACAGTCGCGCAGCATCACGAAGCGCTGGTGGGTCAT
GGGTTTACACATGCCCACATCGTAGCCTTGTCGCAGCACCCTGCAGCCCTTGGCACGGTCGCCGTCAAGT
ACCAGGACATGATTGCGGCGTTGCCGGAAGCCACACATGAGGCGATCGTCGGTGTGGGGAAACAGTGG
AGCGGAGCCCGAGCGCTTGAGGCCCTGTTGACGGTCGCGGGAGAGCTGAGAGGGCCTCCCCTTCAGCT
GGACACGGGCCAGTTGCTGAAGATCGCGAAGCGGGGAGGAGTCACGGCGGTCGAGGCGGTGCACGCG
TGGCGCAATGCGCTCACGGGAGCACCCCTCAACAGTTCACGCTGACAGAGACCGCGGCCGCATTAGGCA
CCCCAGGCTTTACACTTTATGCTTCCGGCTCGTATAATGTGTGGATTTTGAGTTAGGATCCGTCGAGATTT
TCAGGAGCTAAGGAAGCTAAAATGGAGAAAAAAATCACTGGATATACCACCGTTGATATATCCCAATGG
CATCGTAAAGAACATTTTGAGGCATTTCAGTCAGTTGCTCAATGTACCTATAACCAGACCGTTCAGCTGGA
TATTACGGCCTTTTTAAAGACCGTAAAGAAAAATAAGCACAAGTTTTATCCGGCCTTTATTCACATTCTTG
CCCGCCTGATGAATGCTCATCCGGAATTCCGTATGGCAATGAAAGACGGTGAGCTGGTGATATGGGATA
GTGTTCACCCTTGTTACACCGTTTTCCATGAGCAAACTGAAACGTTTTCATCGCTCTGGAGTGAATACCAC
GACGATTTCCGGCAGTTTCTACACATATATTCGCAAGATGTGGCGTGTTACGGTGAAAACCTGGCCTATT
TCCCTAAAGGGTTTATTGAGAATATGTTTTTCGTCTCAGCCAATCCCTGGGTGAGTTTCACCAGTTTTGATT
TAAACGTGGCCAATATGGACAACTTCTTCGCCCCCGTTTTCACCATGGGCAAATATTATACGCAAGGCGA
CAAGGTGCTGATGCCGCTGGCGATTCAGGTTCATCATGCCGTTTGTGATGGCTTCCATGTCGGCAGAATG
CTTAATGAATTACAACAGTACTGCGATGAGTGGCAGGGCGGGGCGTAAAGATCTGGATCCGGCTTACTA
AAAGCCAGATAACAGTATGCGTATTTGCGCGCTGATTTTTGCGGTATAAGAATATATACTGATATGTATA
CCCGAAGTATGTCAAAAAGAGGTATGCTATGAAGCAGCGTATTACAGTGACAGTTGACAGCGACAGCTA
TCAGTTGCTCAAGGCATATATGATGTCAATATCTCCGGTCTGGTAAGCACAACCATGCAGAATGAAGCCC

GTCGTCTGCGTGCCGAACGCTGGAAAGCGGAAAATCAGGAAGGGATGGCTGAGGTCGCCCGGTTTATT
GAAATGAACGGCTCTTTTGCTGACGAGAACAGGGGCTGGTGAAATGCAGTTTAAGGTTTACACCTATAA
AAGAGAGAGCCGTTATCGTCTGTTTGTGGATGTACAGAGTGATATTATTGACACGCCCGGGCGACGGAT
GGTGATCCCCCTGGCCAGTGCACGTCTGCTGTCAGATAAAGTCTCCCGTGAACTTTACCCGGTGGTGCAT
ATCGGGGATGAAAGCTGGCGCATGATGACCACCGATATGGCCAGTGTGCCGGTCTCCGTTATCGGGGAA
GAAGTGGCTGATCTCAGCCACCGCGAAAATGACATCAAAAACGCCATTAACCTGATGTTCTGGGGAATAT
AAATGTCAGGCTCCCTTATACACAGCCAGTCTGCAGGTCGACGGTCTCGCTCTTCGAAGGTTACTTCCCGT
CCTCTGTCAAGCGCACGGCCTCACTCCAGAGCAAGTGGTTGCGATCGCTTCAAACAACGGTGGAAGACCT
GCCCTGGAATCAATCGTGGCCCAGCTTTCGAGGCCGGACCCCGCGCTGGCCGCACTCACTAATGATCATC
TTGTAGCGCTGGCCTGCCTCGGCGGACGACCCGCCTTGGATGCGGTGAAGAAGGGGCTCCCGCACGCGC
CTGCATTGATTAAGCGGACCAACAGAAGGATTCCCGAGAGGACATAGCCCCAAGAAGAAGAGAAAGGT
GGAGGCCAGCGGTTCCGGACGGGCTGACGCATTGGACGATTTTGATCTGGATATGCTGGGAAGTGACG
CCCTCGATGATTTTGACCTTGACATGCTTGGTTCGGATGCCCTTGATGACTTTGACCTCGACATGCTCGGC
AGTGACGCCCTTGATGATTTCGACCTGGACATGCTGATTAACTCTAGAGGCAGTGGAGAGGGCAGAGGA
AGTCTGCTAACATGCGGTGACGTCGAGGAGAATCCTGGCCCAGTGAGCAAGGGCGAGGAGCTGTTCAC
CGGGGTGGTGCCCATCCTGGTCGAGCTGGACGGCGACGTAAACGGCCACAAGTTCAGCGTGTCCGGCG
AGGGCGAGGGCGATGCCACCTACGGCAAGCTGACCCTGAAGTTCATCTGCACCACCGGCAAGCTGCCCG
TGCCCTGGCCCACCCTCGTGACCACCCTGACCTACGGCGTGCAGTGCTTCAGCCGCTACCCCGACCACAT
GAAGCAGCACGACTTCTTCAAGTCCGCCATGCCCGAAGGCTACGTCCAGGAGCGCACCATCTTCTTCAAG
GACGACGGCAACTACAAGACCCGCGCCGAGGTGAAGTTCGAGGGCGACACCCTGGTGAACCGCATCGA
GCTGAAGGGCATCGACTTCAAGGAGGACGGCAACATCCTGGGGCACAAGCTGGAGTACAACTACAACA
GCCACAACGTCTATATCATGGCCGACAAGCAGAAGAACGGCATCAAGGTGAACTTCAAGATCCGCCACA
ACATCGAGGACGGCAGCGTGCAGCTCGCCGACCACTACCAGCAGAACACCCCCATCGGCGACGGCCCCG
TGCTGCTGCCCGACAACCACTACCTGAGCACCCAGTCCGCCCTGAGCAAAGACCCCAACGAGAAGCGCG
ATCACATGGTCCTGCTGGAGTTCGTGACCGCCGCCGGGATCACTCTCGGCATGGACGAGCTGTACAAG

Supplementary Sequence 2

gRNA backbone sequence

AflII cloning site

AGCGCCCAATACGCAAACCGCCTCTCCCCGCGCGTTGGCCGATTCATTAATGCAGCTGGCACGACAGGTT
TCCCGACTGGAAAGCGGGCAGTGAGCGCAACGCAATTAATGTGAGTTAGCTCACTCATTAGGCACCCCA
GGCTTTACACTTTATGCTTCCGGCTCGTATGTTGTGTGGAATTGTGAGCGGATAACAATTTCACACAGGA
AACAGCTATGACCATGATTACGCCAAGCTATTTAGGTGACACTATAGAATACTCAAGCTATGCATCAAGC
TTGGTACCGAGCTCGGATCCACTAGTAACGGCCGCCAGTGTGCTGGAATTCGCCCTTAAGGGCGAATTCT
GCAGATATCCATCACACTGGCGGCCGCTCGAGCATGCATCTAGAGGGCCCAATTCGCCCTATAGTGAGTC
GTATTACAATTCACTGGCCGTCGTTTTACAACGTCGTGACTGGGAAAACCCTGGCGTTACCCAACTTAATC
GCCTTGCAGCACATCCCCCTTTCGCCAGCTGGCGTAATAGCGAAGAGGCCCGCACCGATCGCCCTTCCCA
ACAGTTGCGCAGCCTATACGTACGGCAGTTTAAGGTTTACACCTATAAAAGAGAGAGCCGTTATCGTCTG
TTTGTGGATGTACAGAGTGATATTATTGACACGCCGGGGCGACGGATGGTGATCCCCCTGGCCAGTGCA

CGTCTGCTGTCAGATAAAGTCTCCCGTGAACTTTACCCGGTGGTGCATATCGGGGATGAAAGCTGGCGC
ATGATGACCACCGATATGGCCAGTGTGCCGGTCTCCGTTATCGGGGAAGAAGTGGCTGATCTCAGCCAC
CGCGAAAATGACATCAAAAACGCCATTAACCTGATGTTCTGGGGAATATAAATGTCAGGCATGAGATTAT
CAAAAAGGATCTTCACCTAGATCCTTTTCACGTAGAAAGCCAGTCCGCAGAAACGGTGCTGACCCCGGAT
GAATGTCAGCTACTGGGCTATCTGGACAAGGGAAAACGCAAGCGCAAAGAGAAAGCAGGTAGCTTGCA
GTGGGCTTACATGGCGATAGCTAGACTGGGCGGTTTTATGGACAGCAAGCGAACCGGAATTGCCAGCTG
GGGCGCCCTCTGGTAAGGTTGGGAAGCCCTGCAAAGTAAACTGGATGGCTTTCTCGCCGCCAAGGATCT
GATGGCGCAGGGGATCAAGCTCTGATCAAGAGACAGGATGAGGATCGTTTCGCATGATTGAACAAGAT
GGATTGCACGCAGGTTCTCCGGCCGCTTGGGTGGAGAGGCTATTCGGCTATGACTGGGCACAACAGACA
ATCGGCTGCTCTGATGCCGCCGTGTTCCGGCTGTCAGCGCAGGGGCGCCCGGTTCTTTTTGTCAAGACCG
ACCTGTCCGGTGCCCTGAATGAACTGCAAGACGAGGCAGCGCGGCTATCGTGGCTGGCCACGACGGGC
GTTCCTTGCGCAGCTGTGCTCGACGTTGTCACTGAAGCGGGAAGGGACTGGCTGCTATTGGGCGAAGTG
CCGGGGCAGGATCTCCTGTCATCTCACCTTGCTCCTGCCGAGAAAGTATCCATCATGGCTGATGCAATGC
GGCGGCTGCATACGCTTGATCCGGCTACCTGCCCATTCGACCACCAAGCGAAACATCGCATCGAGCGAG
CACGTACTCGGATGGAAGCCGGTCTTGTCGATCAGGATGATCTGGACGAAGAGCATCAGGGGCTCGCGC
CAGCCGAACTGTTCGCCAGGCTCAAGGCGAGCATGCCCGACGGCGAGGATCTCGTCGTGACCCATGGCG
ATGCCTGCTTGCCGAATATCATGGTGGAAAATGGCCGCTTTTCTGGATTCATCGACTGTGGCCGGCTGGG
TGTGGCGGACCGCTATCAGGACATAGCGTTGGCTACCCGTGATATTGCTGAAGAGCTTGGCGGCGAATG
GGCTGACCGCTTCCTCGTGCTTTACGGTATCGCCGCTCCCGATTCGCAGCGCATCGCCTTCTATCGCCTTC
TTGACGAGTTCTTCTGAATTATTAACGCTTACAATTTCCTGATGCGGTATTTTCTCCTTACGCATCTGTGCG
GTATTTCACACCGCATACAGGTGGCACTTTTCGGGGAAATGTGCGCGGAACCCCTATTTGTTTATTTTTCT
AAATACATTCAAATATGTATCCGCTCATGAGACAATAACCCTGATAAATGCTTCAATAATAGCACGTGAG
GAGGGCCACCATGGCCAAGTTGACCAGTGCCGTTCCGGTGCTCACCGCGCGCGACGTCGCCGGAGCGGT
CGAGTTCTGGACCGACCGGCTCGGGTTCTCCCGGGACTTCGTGGAGGACGACTTCGCCGGTGTGGTCCG
GGACGACGTGACCCTGTTCATCAGCGCGGTCCAGGACCAGGTGGTGCCGGACAACACCCTGGCCTGGGT
GTGGGTGCGCGGCCTGGACGAGCTGTACGCCGAGTGGTCGGAGGTCGTGTCCACGAACTTCCGGGACG
CCTCCGGGCCGGCCATGACCGAGATCGGCGAGCAGCCGTGGGGGCGGGAGTTCGCCCTGCGCGACCCG
GCCGGCAACTGCGTGCACTTCGTGGCCGAGGAGCAGGACTGACACGTGCTAAAACTTCATTTTTAATTTA
AAAGGATCTAGGTGAAGATCCTTTTTGATAATCTCATGACCAAAATCCCTTAACGTGAGTTTTCGTTCCAC
TGAGCGTCAGACCCCGTAGAAAAGATCAAAGGATCTTCTTGAGATCCTTTTTTTCTGCGCGTAATCTGCTG
CTTGCAAACAAAAAAACCACCGCTACCAGCGGTGGTTTGTTTGCCGGATCAAGAGCTACCAACTCTTTTTC
CGAAGGTAACTGGCTTCAGCAGAGCGCAGATACCAAATACTGTCCTTCTAGTGTAGCCGTAGTTAGGCCA
CCACTTCAAGAACTCTGTAGCACCGCCTACATACCTCGCTCTGCTAATCCTGTTACCAGTGGCTGCTGCCA
GTGGCGATAAGTCGTGTCTTACCGGGTTGGACTCAAGACGATAGTTACCGGATAAGGCGCAGCGGTCGG
GCTGAACGGGGGGTTCGTGCACACAGCCCAGCTTGGAGCGAACGACCTACACCGAACTGAGATACCTAC
AGCGTGAGCTATGAGAAAGCGCCACGCTTCCCGAAGGGAGAAAGGCGGACAGGTATCCGGTAAGCGGC
AGGGTCGGAACAGGAGAGCGCACGAGGGAGCTTCCAGGGGGAAACGCCTGGTATCTTTATAGTCCTGT
CGGGTTTCGCCACCTCTGACTTGAGCGTCGATTTTTGTGATGCTCGTCAGGGGGGCGGAGCCTATGGAA
AAACGCCAGCAACGCGGCCTTTTTACGGTTCCTGGGCTTTTGCTGGCCTTTTGCTCACATGTTCTTTCCTGC
GTTATCCCCTGATTCTGTGGATAACCGTATTACCGCCTTTGAGTGAGCTGATACCGCTCGCCGCAGCCGA
ACGACCGAGCGCAGCGAGTCAGTGAGCGAGGAAGCGGAAG

Supplmentary Figure 1

(a)

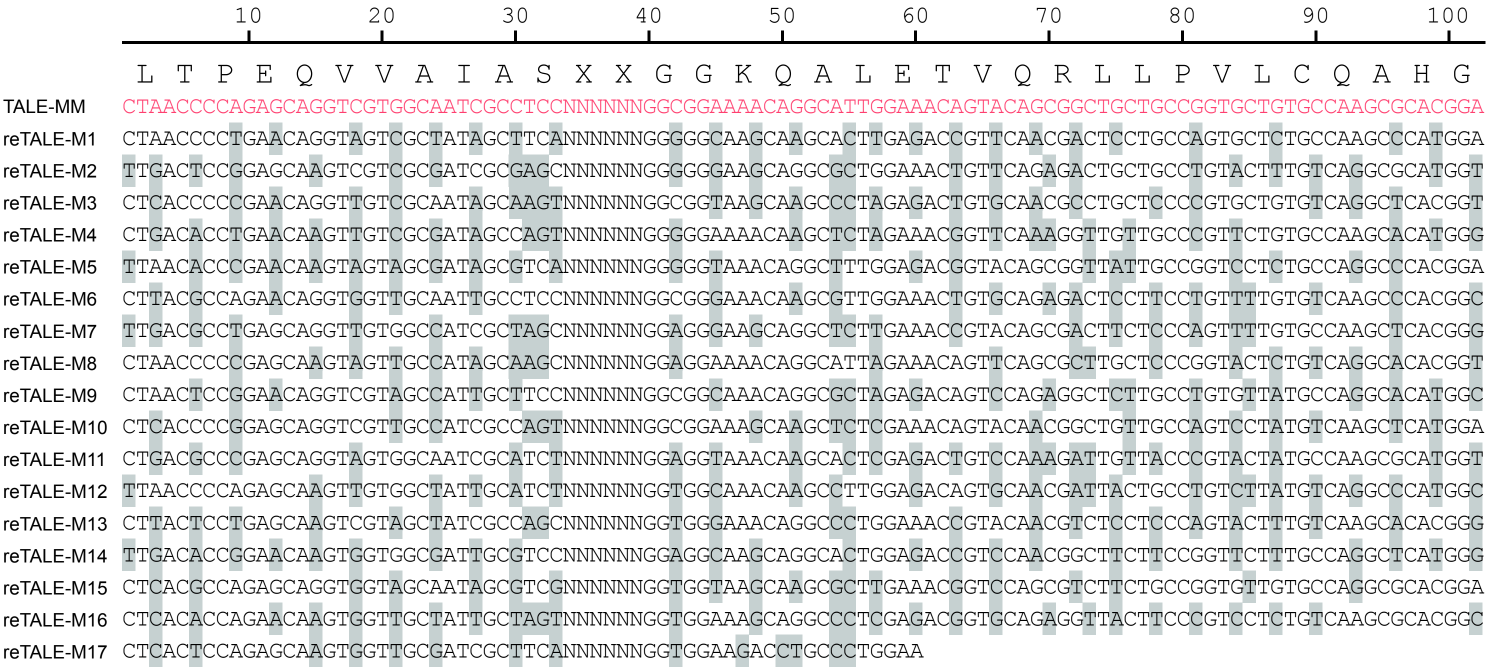

(b)

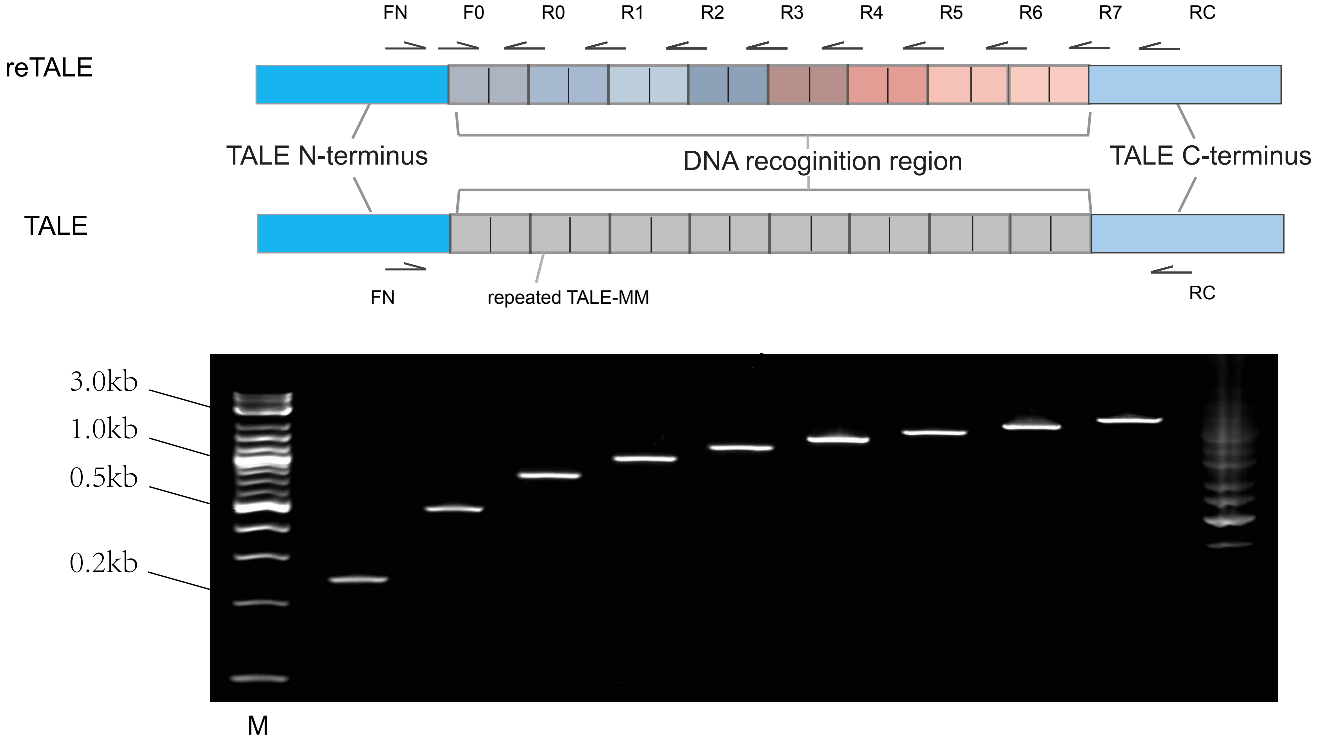

Template	reTALE	reTALE	reTALE	reTALE	reTALE	reTALE	reTALE	reTALE	reTALE	TALE
Primer1	F0	F0	F0	F0	F0	F0	F0	F0	FN	FN
Primer2	R0	R1	R2	R3	R4	R5	R6	R7	RC	RC

Supplmentary Figure 2

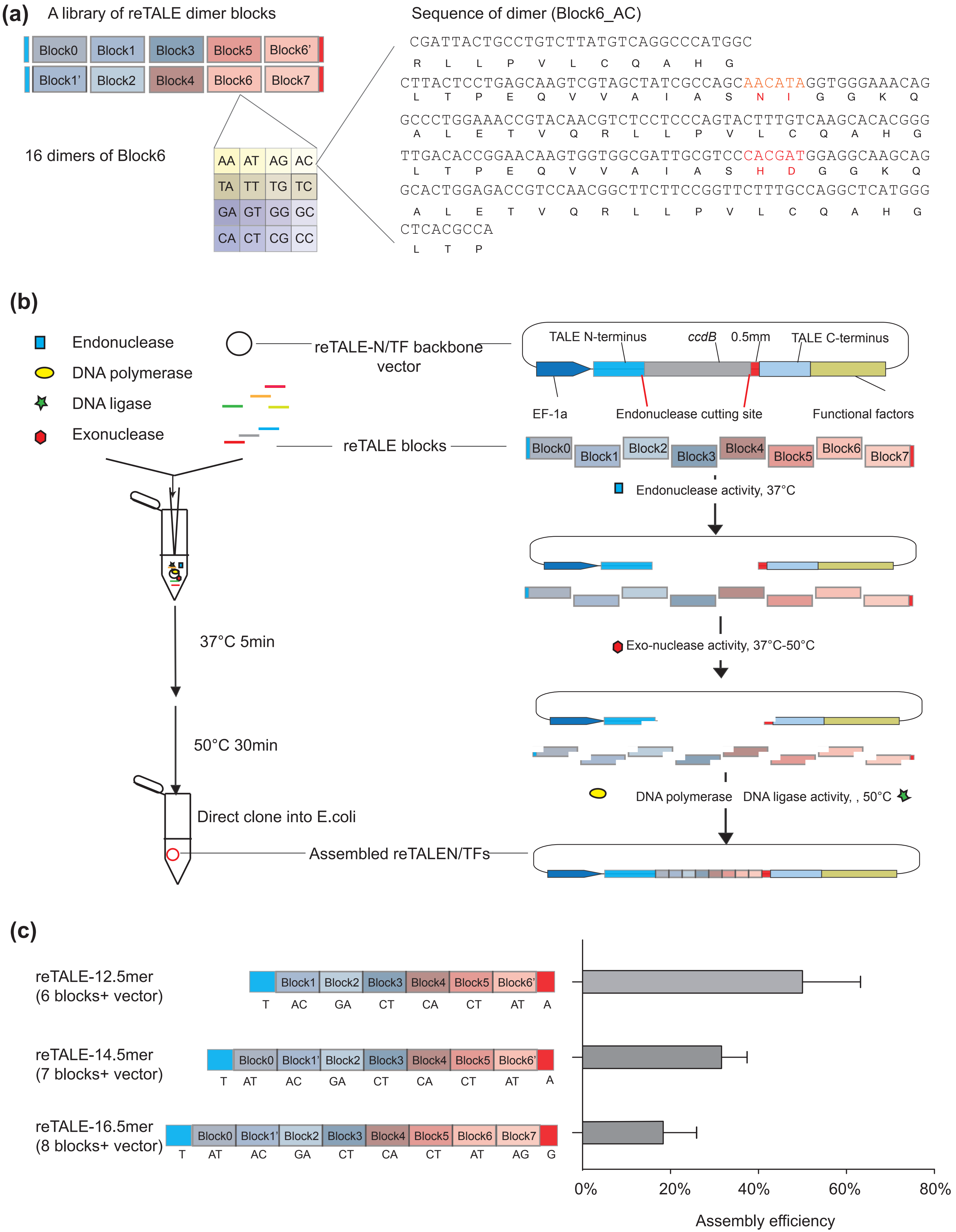

Supplementary Figure 3

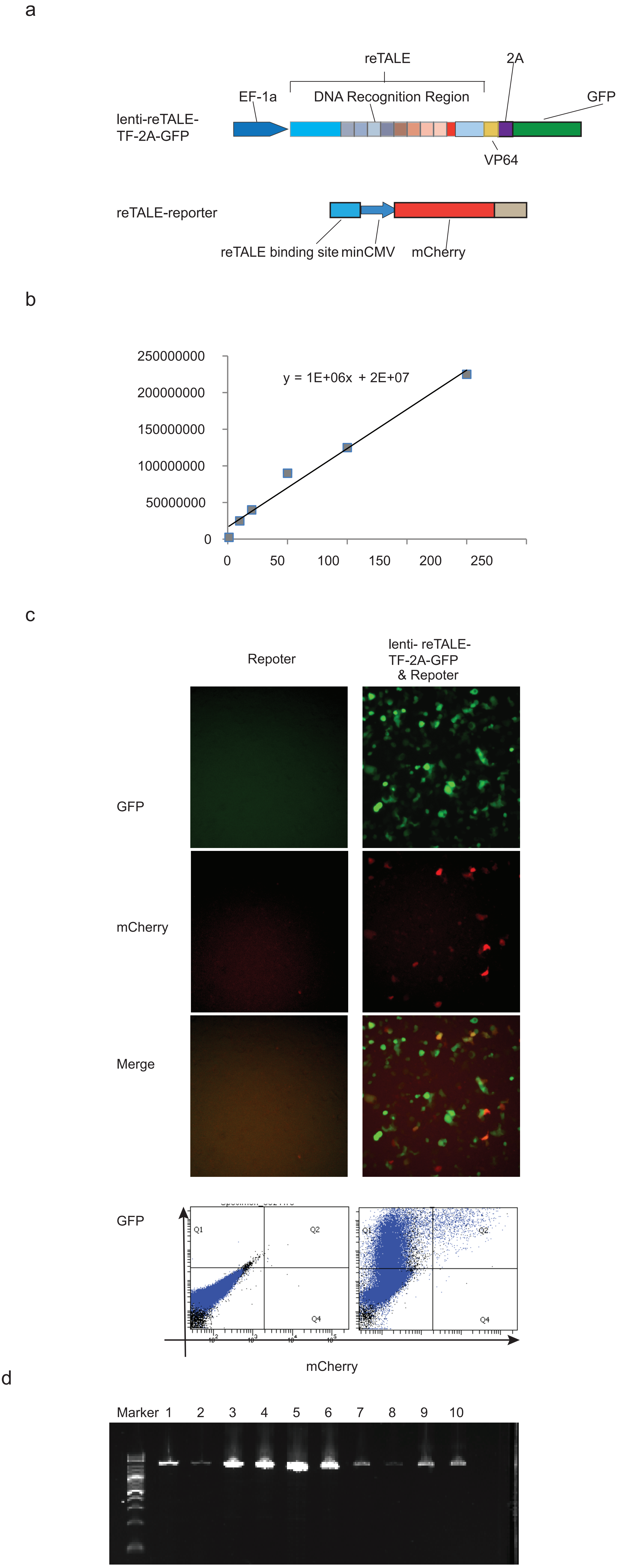

Supplementary Figure 4

(a)

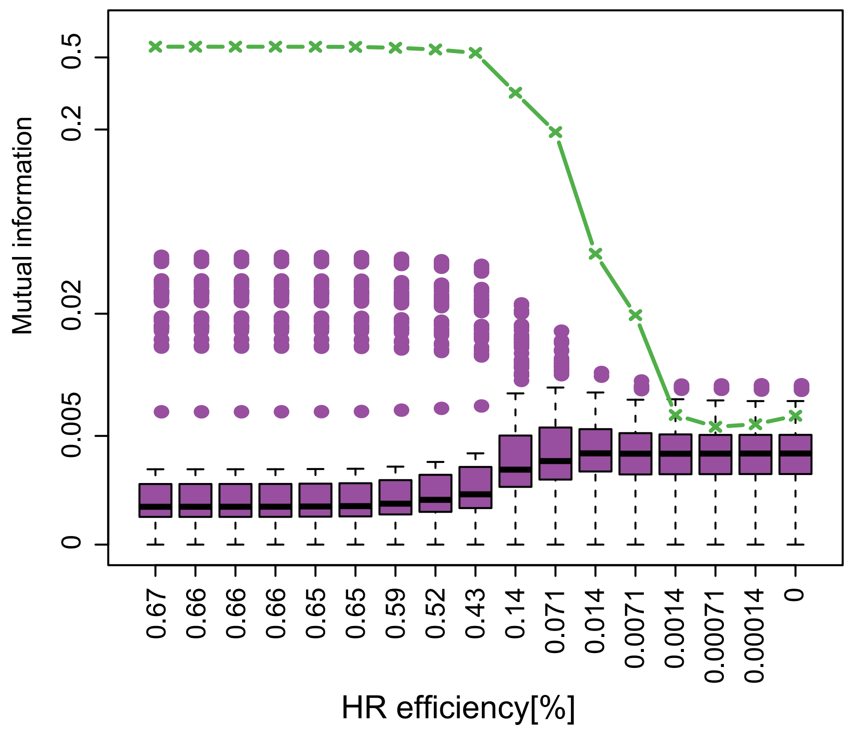

(b)

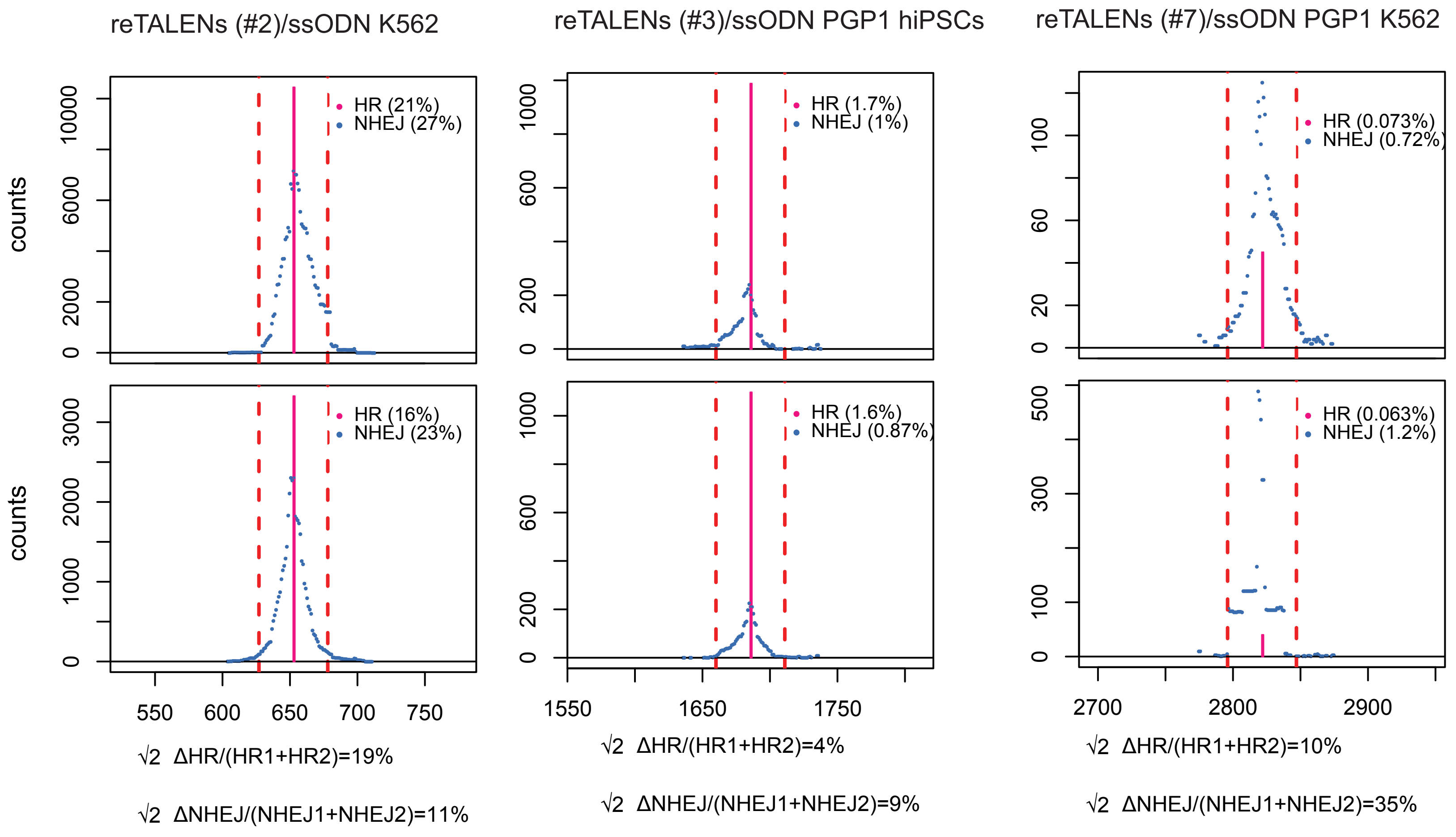

Supplementary Figure 5

(a)

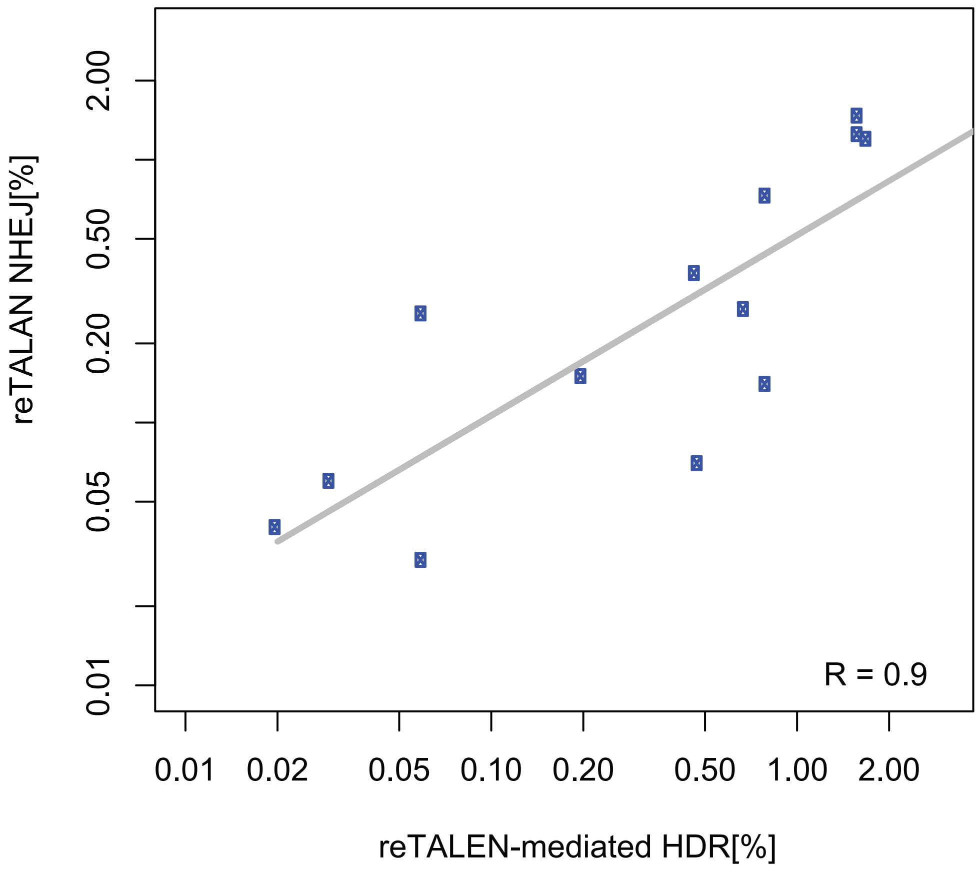

(b)

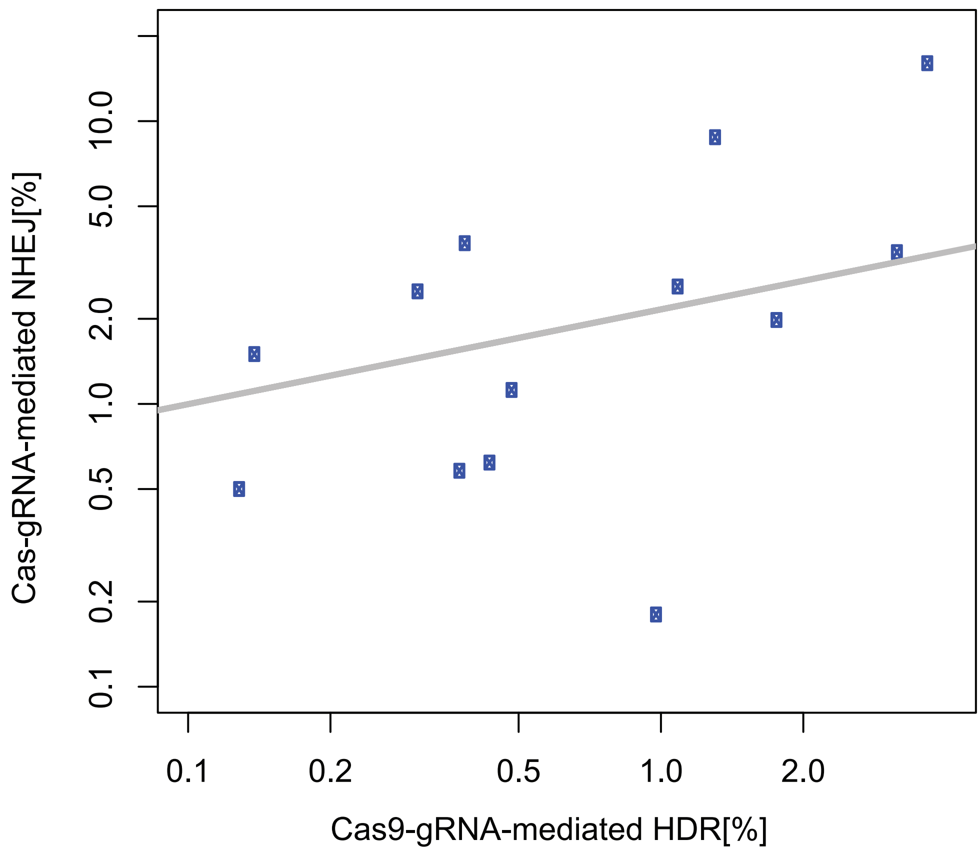

(c)

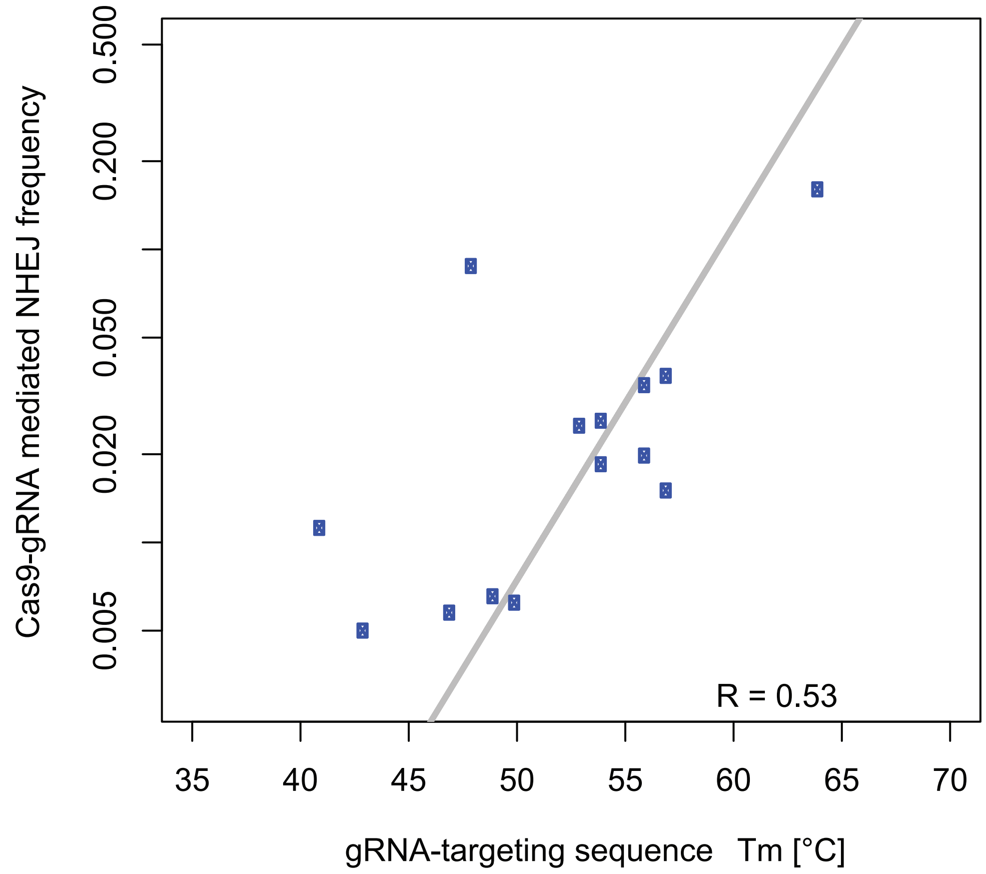

Supplmentary Figure 6

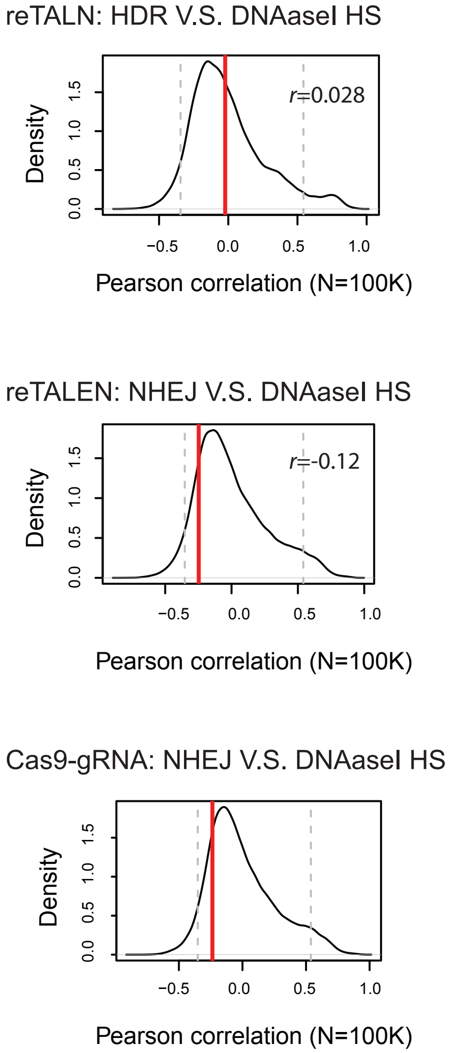

Supplementary Figure 7

(a)

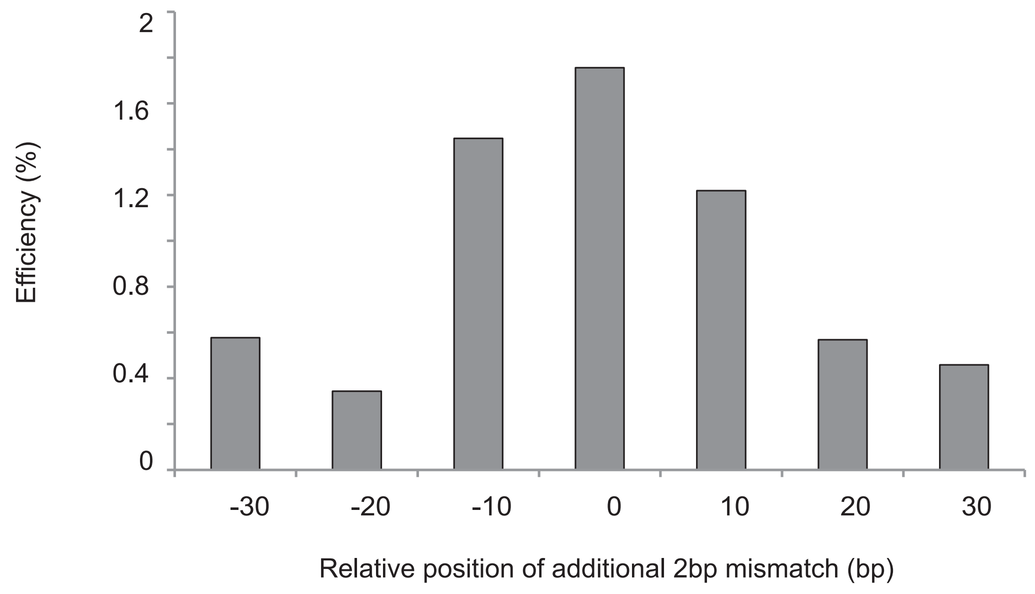

(b)

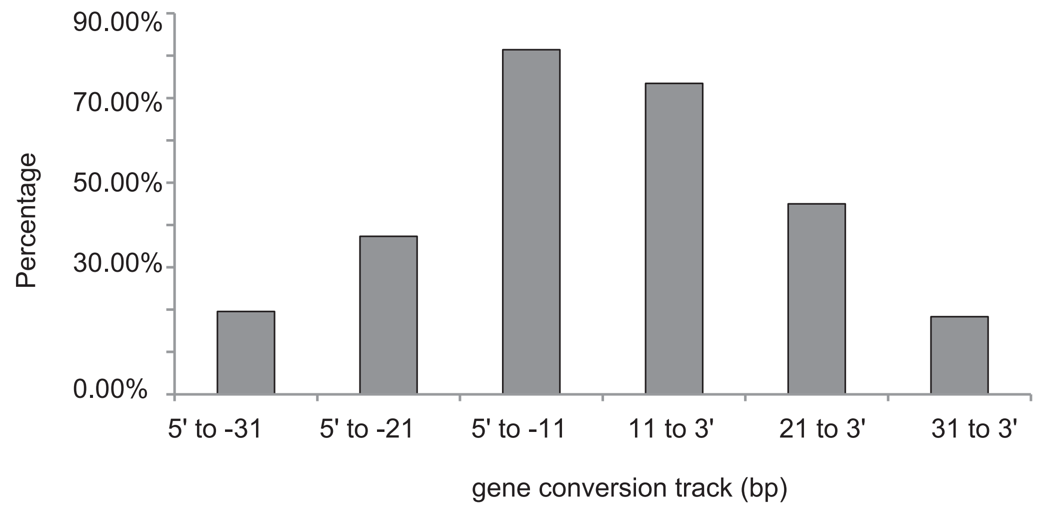

(c)

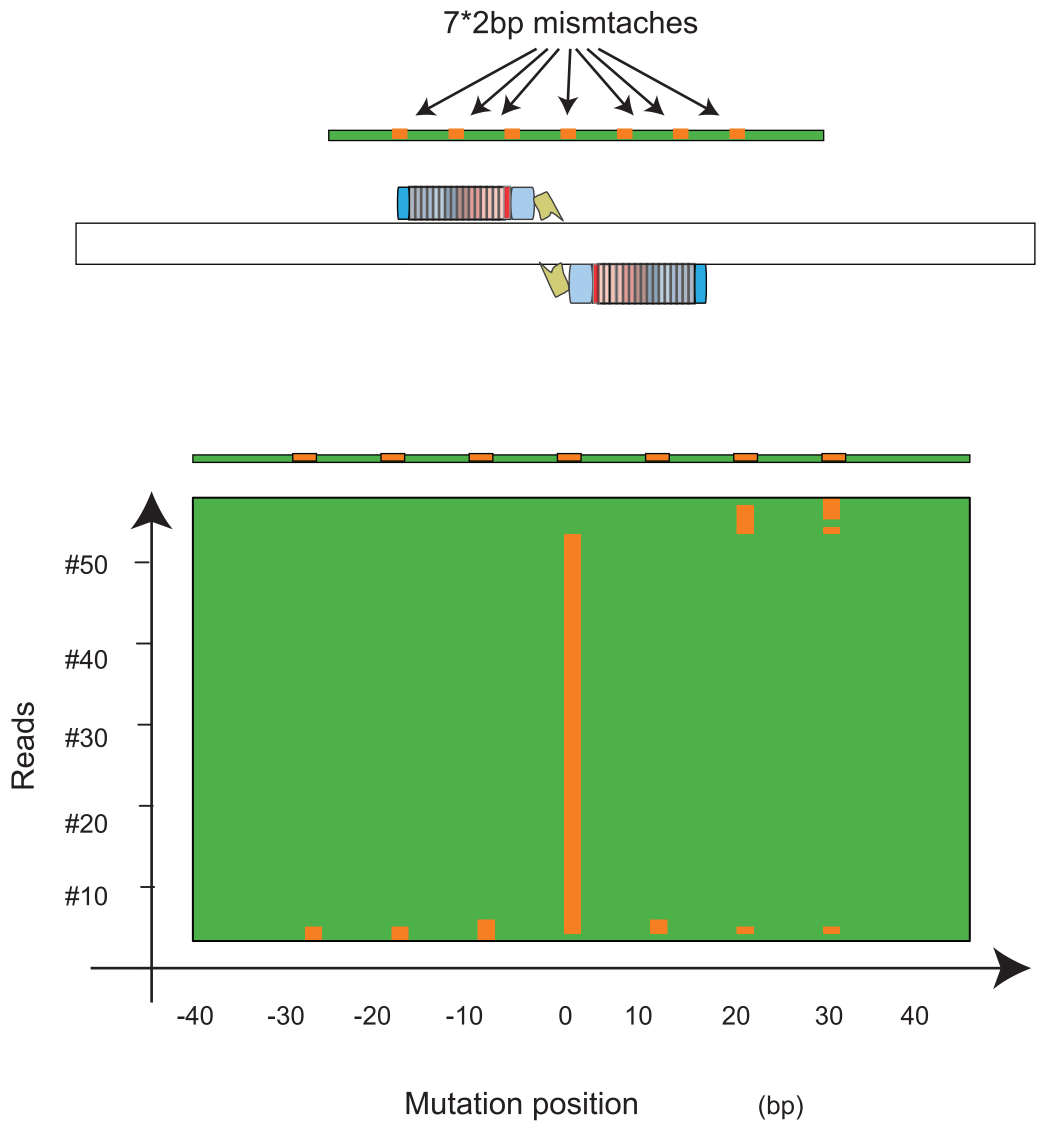

Supplementary Figure 8

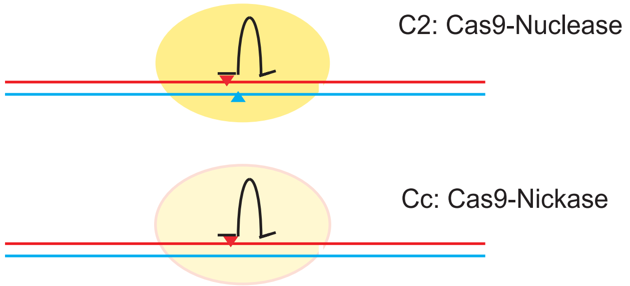

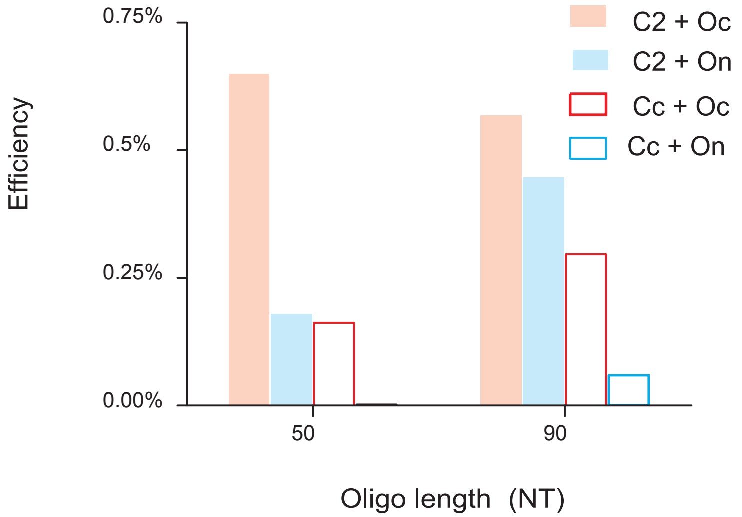

Supplement Figure 9

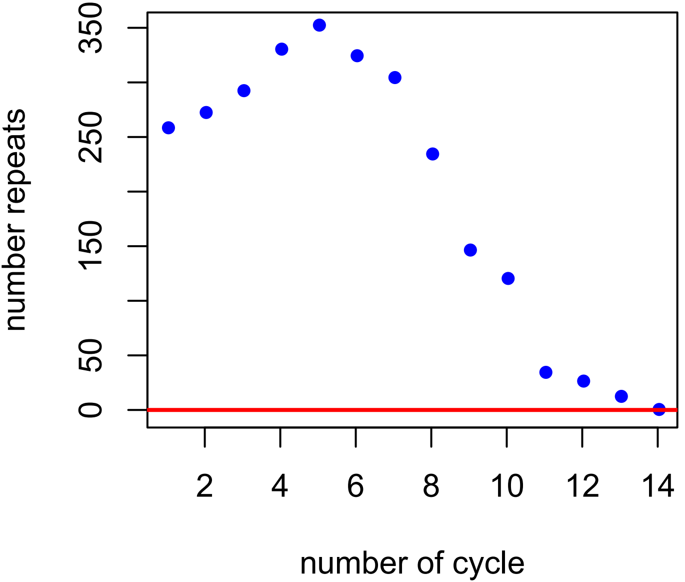

Supplement: Supplementary Data [file supp_gkt555_nar-00873-h-2013-File003.pdf]
